# Supplementary figures and images for: Learning with filopodia and spines: Complementary strong and weak competition lead to specialized, graded, and protected receptive fields
Source: PLoS Comput Biol. 2024 May 14;20(5):e1012110. doi: 10.1371/journal.pcbi.1012110 (PMC11125506; doi:10.1371/journal.pcbi.1012110)

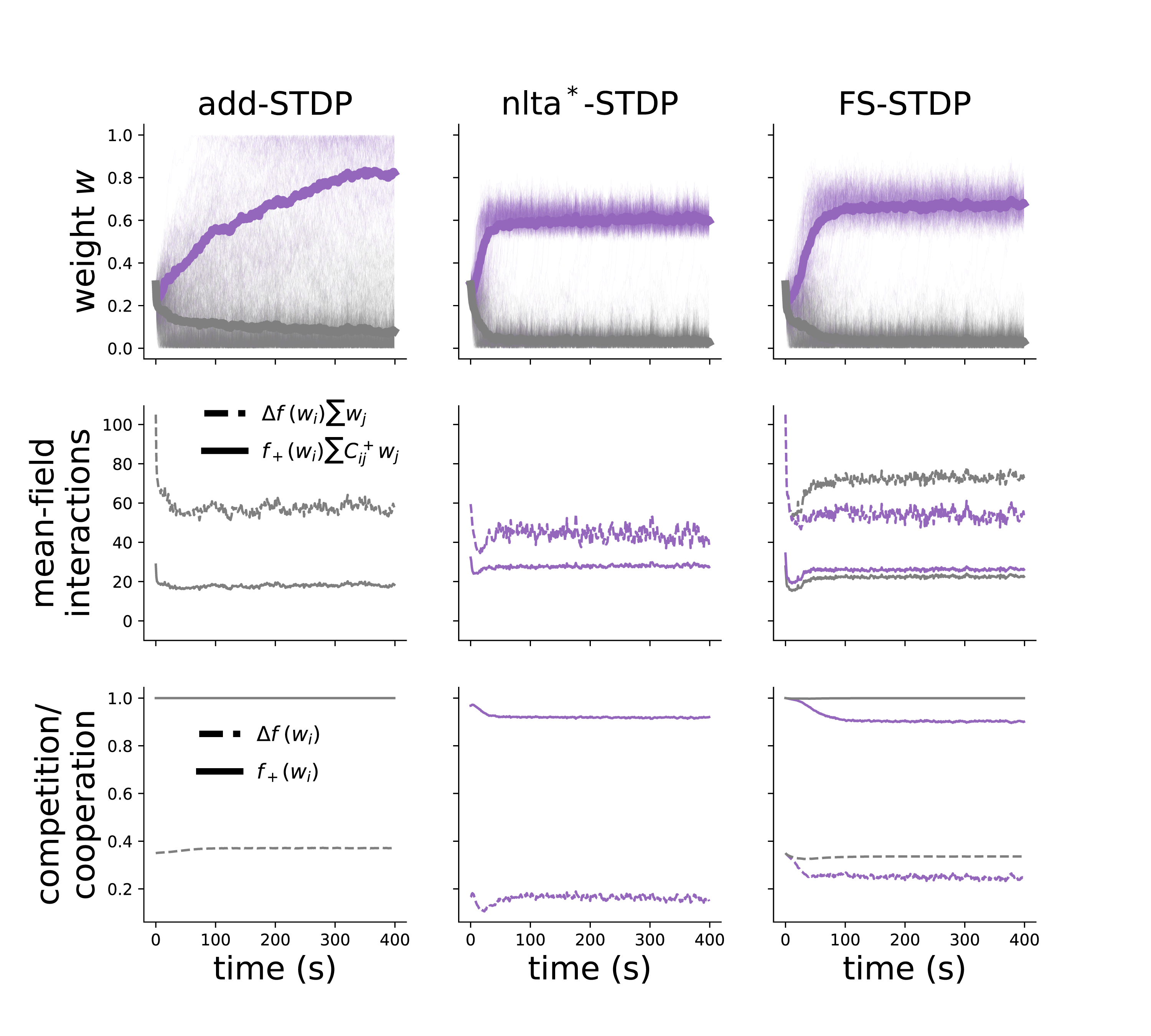

Supplement: S1 Fig — Obtained for add-STDP, nlta*-STDP and FS-STDP (left to right). As usual, gray means filopodia in the case of FS-STDP, and simply wi > 0 for the rest of learning rules. Note how grey curves of FS-STDP approximate those of add-STDP, and purple ones those of nlta*-STDP. μ = 0.91 (for nlta*-STDP) taken as the average μ of spines obtained via FS-STDP. (TIF) [file pcbi.1012110.s001.tif]

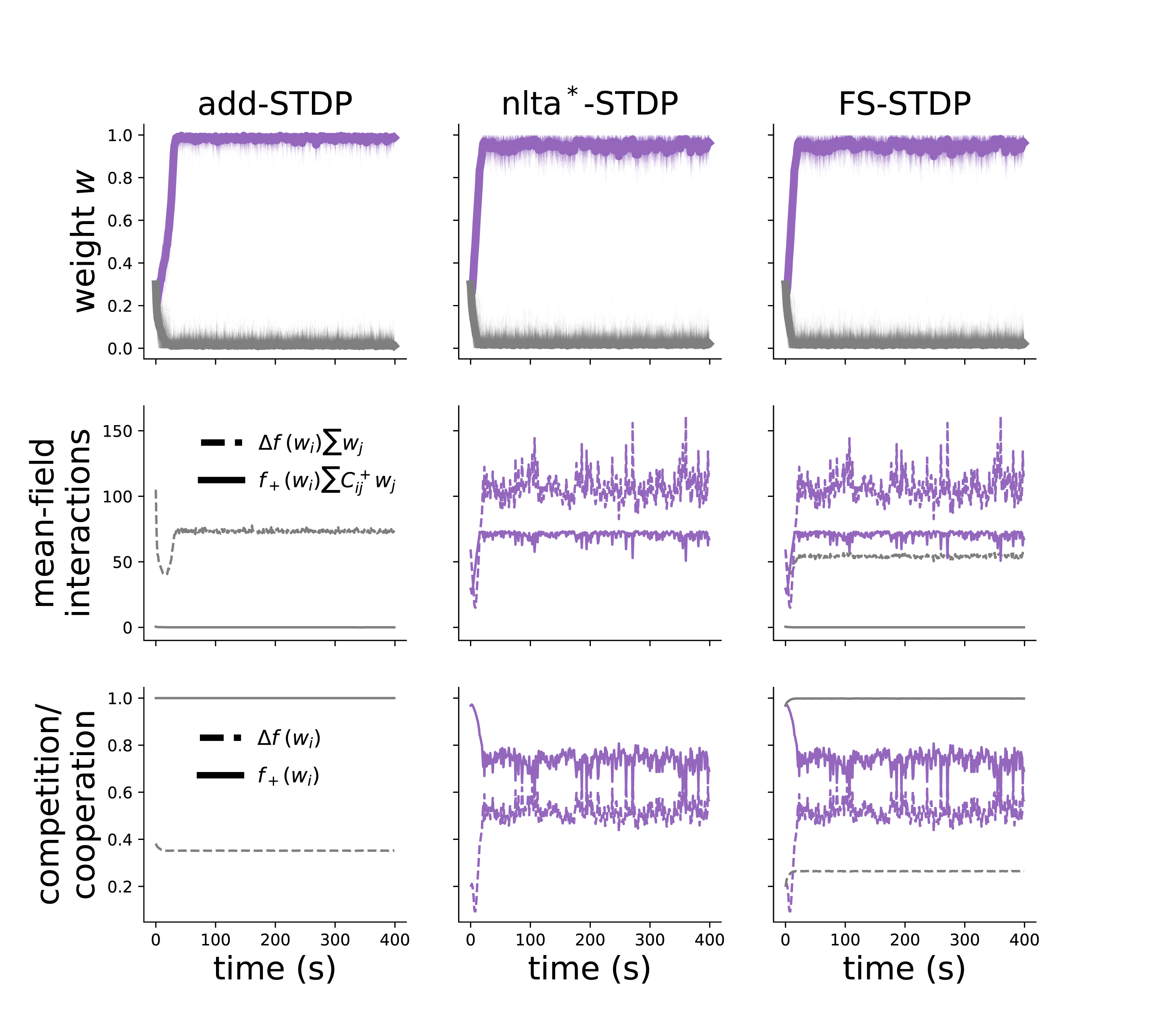

Supplement: S2 Fig — μ = 0.12 (for nlta*-STDP) taken as the average μ of spines obtained via FS-STDP. (TIF) [file pcbi.1012110.s002.tif]

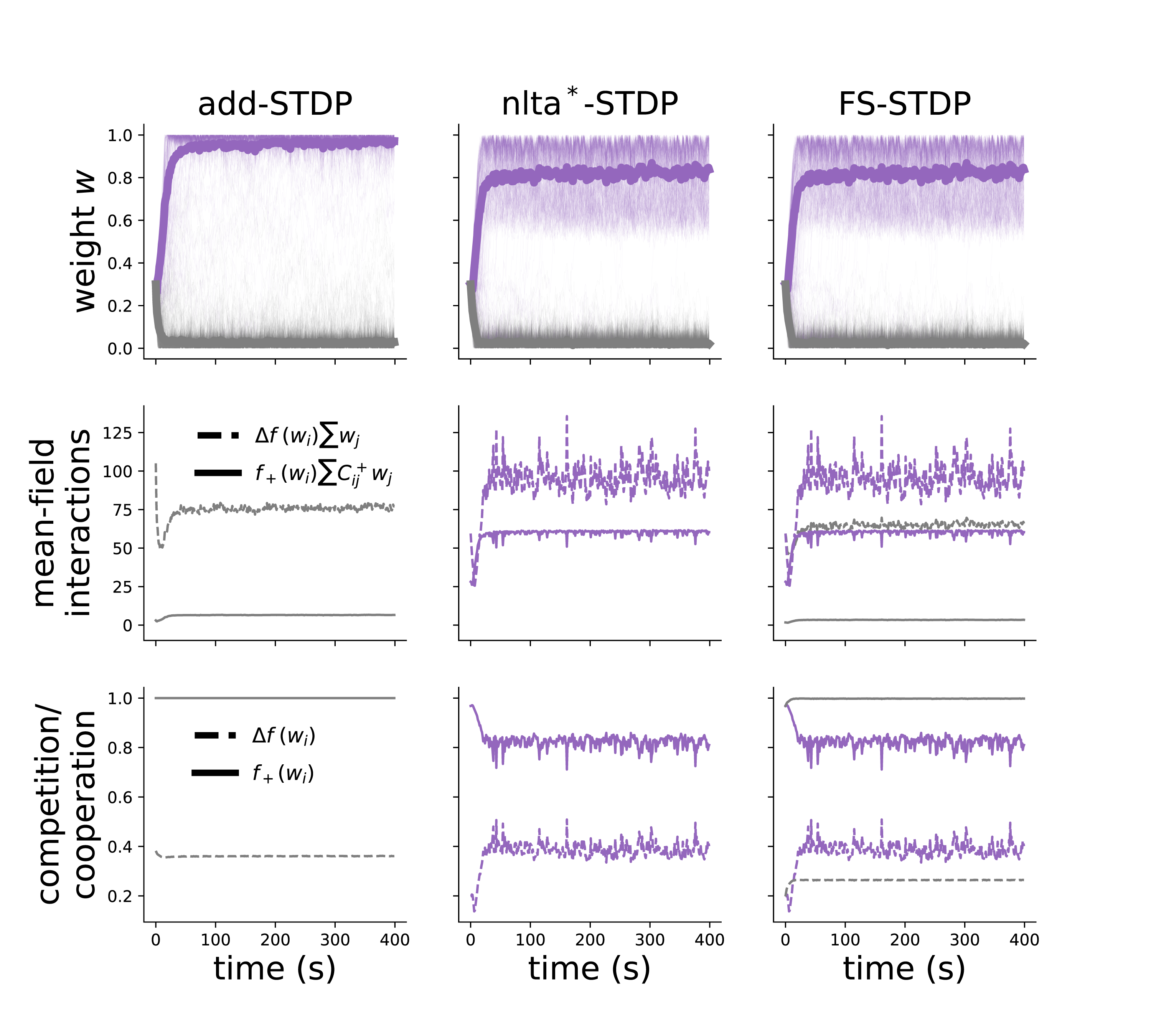

Supplement: S3 Fig — This structure is used throughout the paper for its similarity to a squared pulse (used in most previous studies), its yet rich structure (not exaclty binary) and the plausibility of these type of correlations in visual cortex. μ = 0.11 (for nlta*-STDP) taken as the average μ of spines obtained via FS-STDP. (TIF) [file pcbi.1012110.s003.tif]

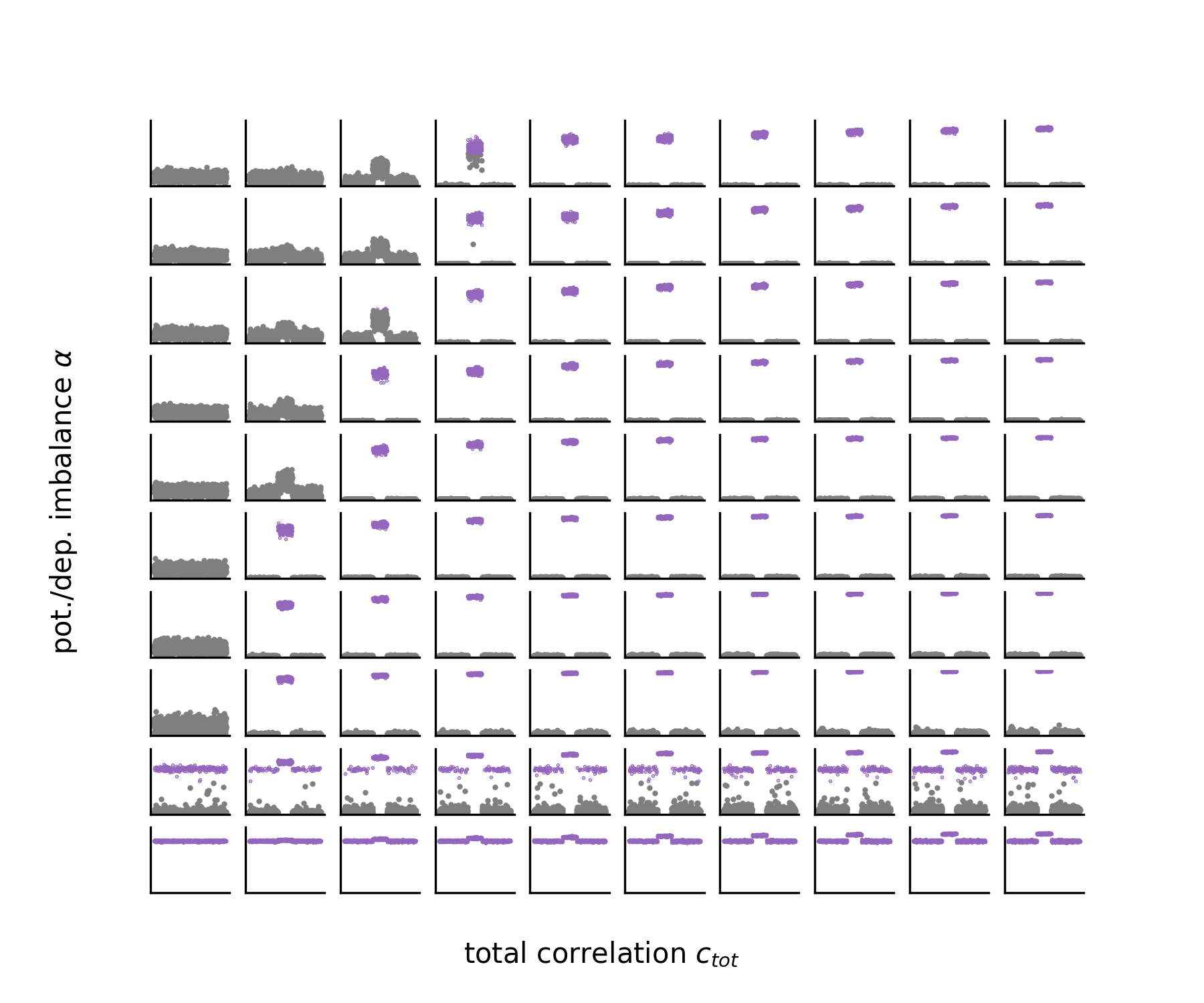

Supplement: S4 Fig — ctot and α as in the 10x10 pixels of heatmaps in Fig 3. (TIF) [file pcbi.1012110.s004.tif]

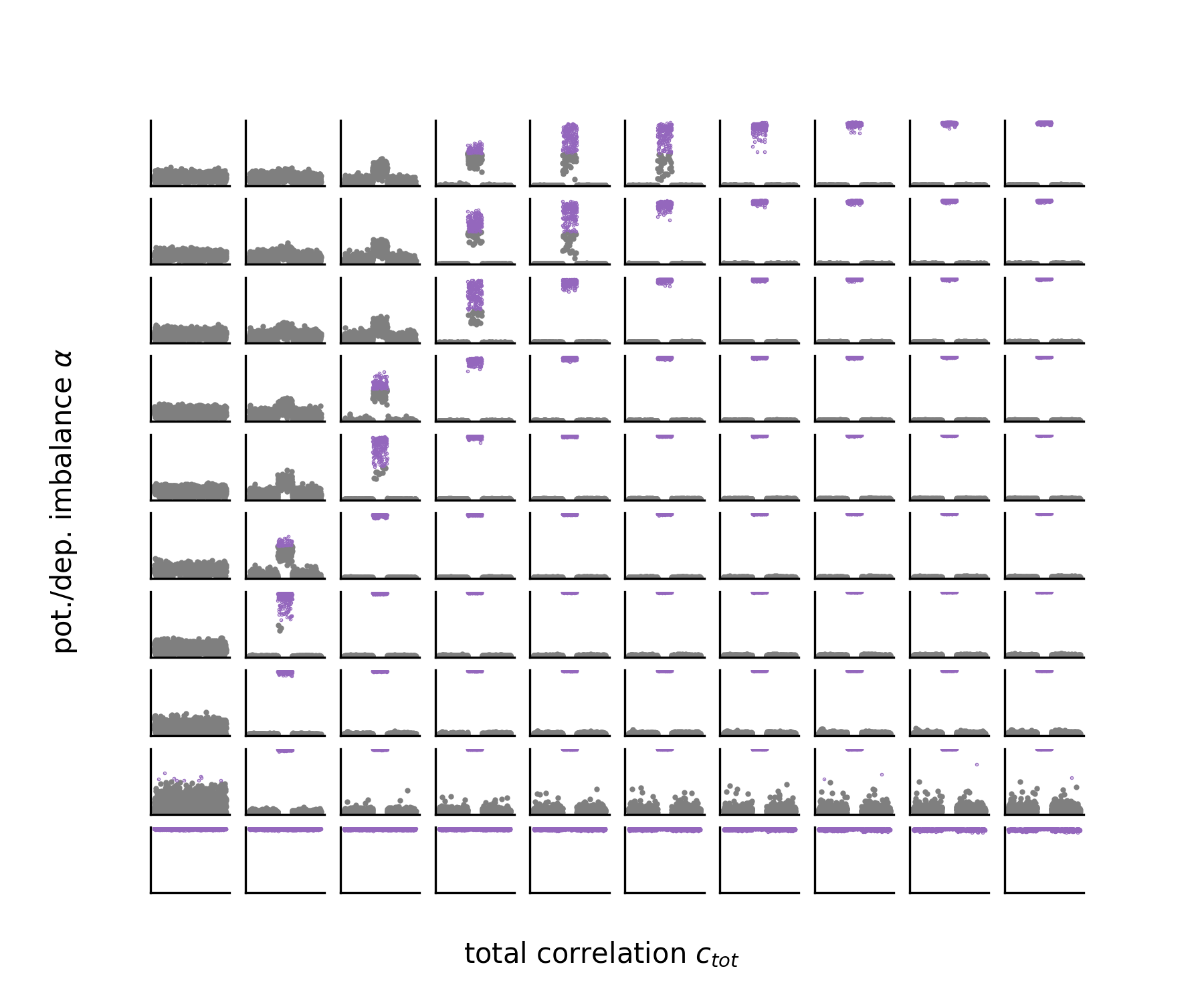

Supplement: S5 Fig — ctot and α as in the 10x10 pixels of heatmaps in Fig 3. (TIF) [file pcbi.1012110.s005.tif]

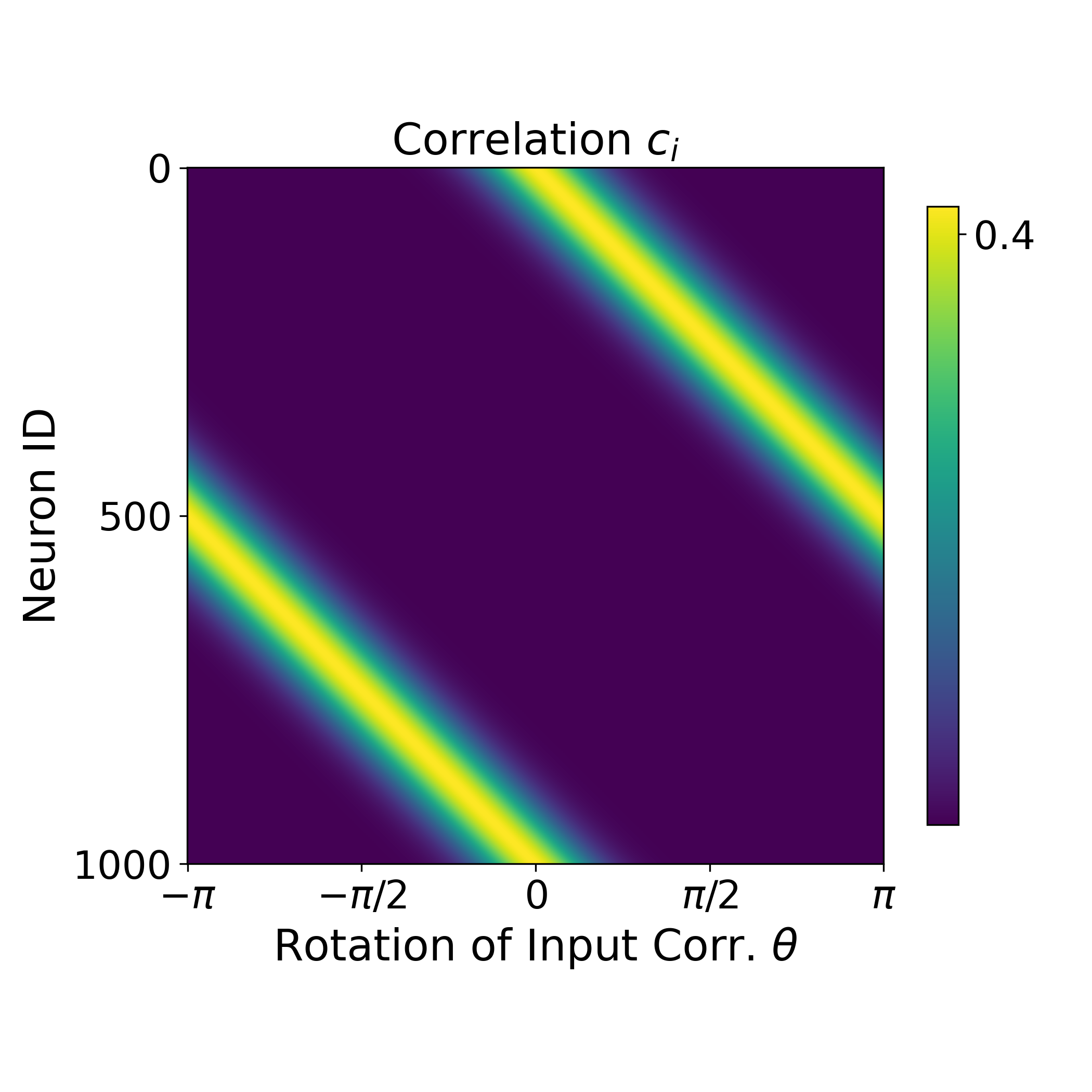

Supplement: S6 Fig — (TIF) [file pcbi.1012110.s006.tif]

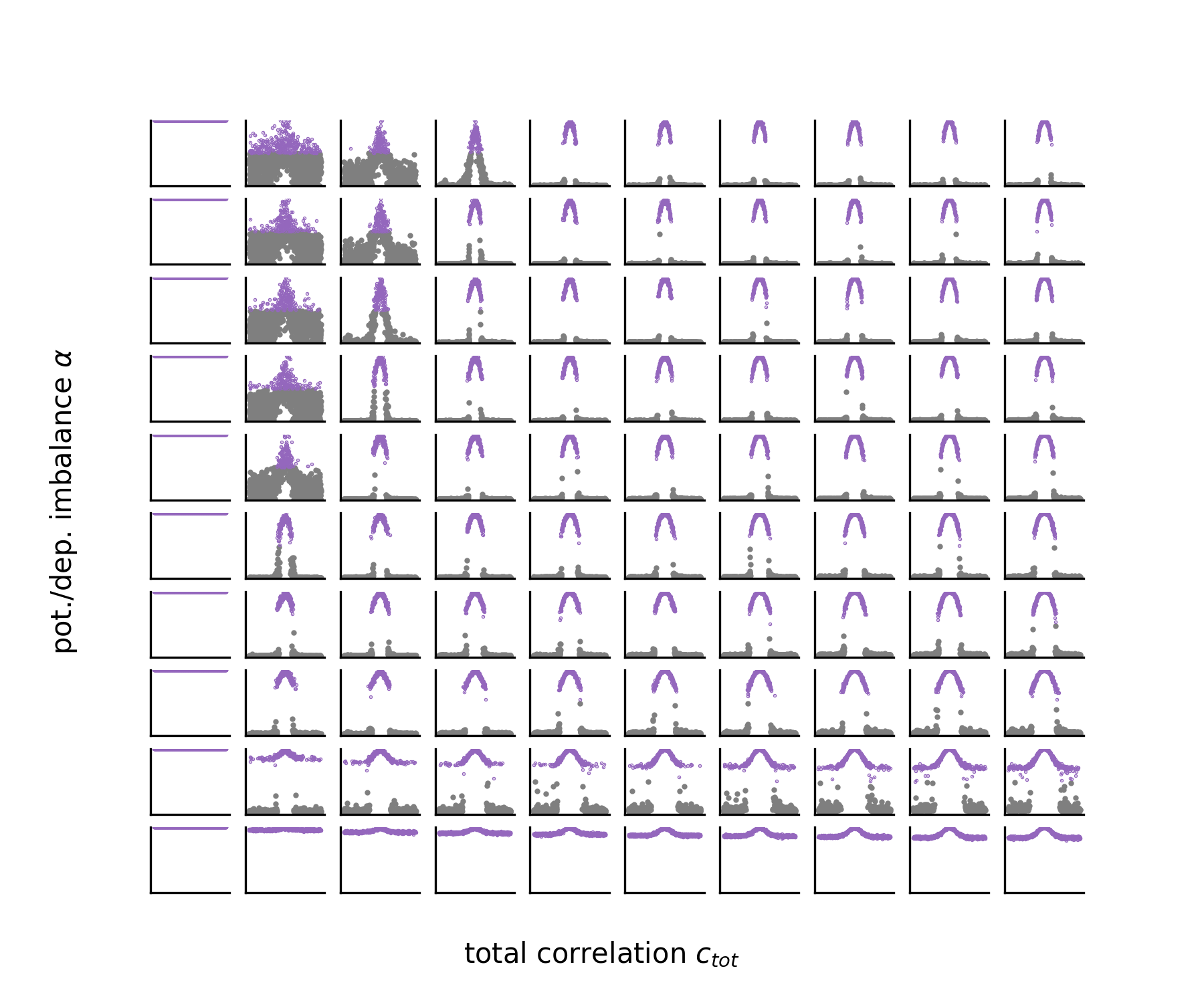

Supplement: S7 Fig — Each subpanel corresponds to each of the 10x10 pixels in the heatmaps shown in Fig 3A1 and 3B1. (TIF) [file pcbi.1012110.s007.tif]

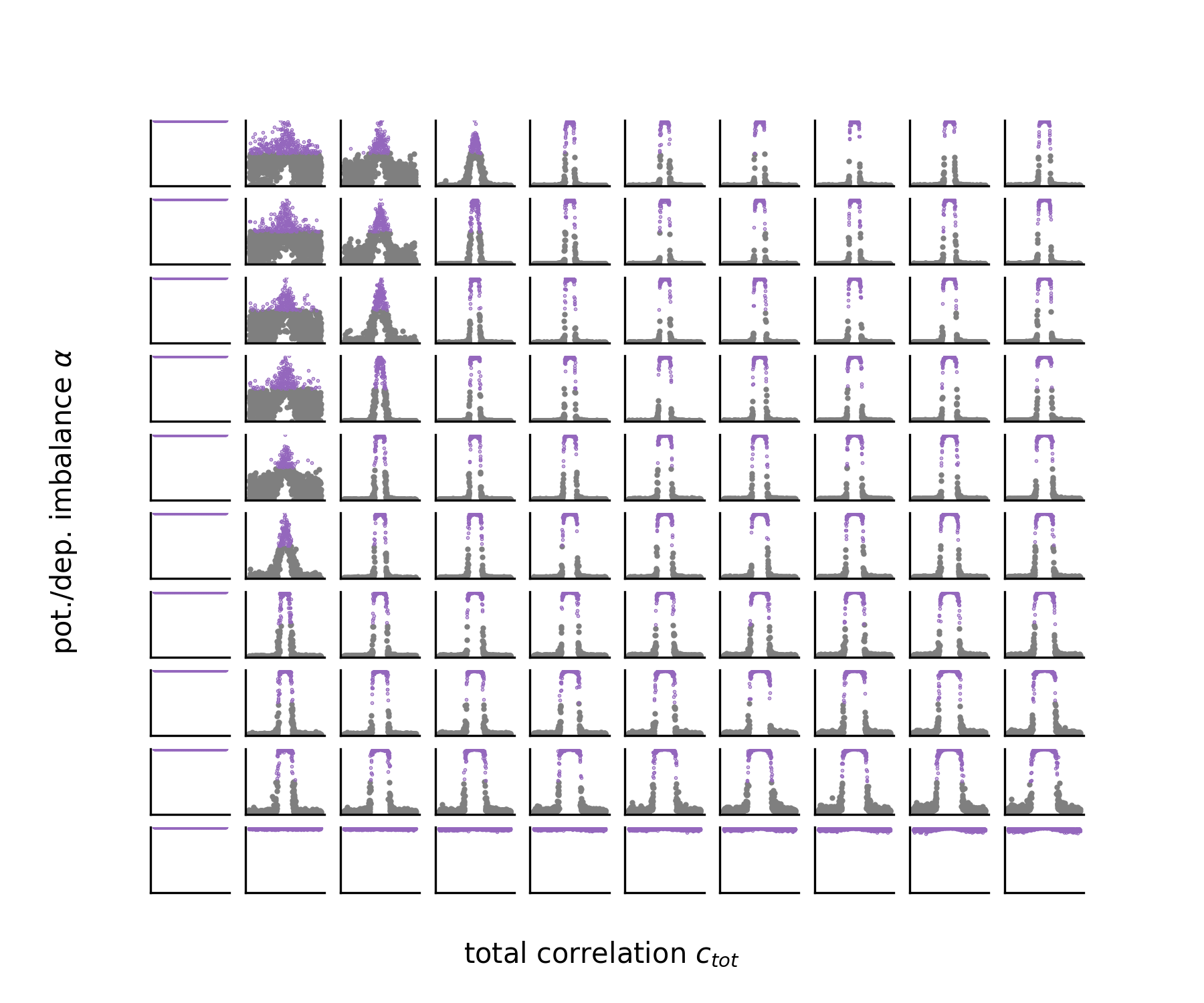

Supplement: S8 Fig — Each subpanel corresponds to each of the 10x10 pixels in the heatmaps shown in Fig 3A2 and 3B2. (TIF) [file pcbi.1012110.s008.tif]

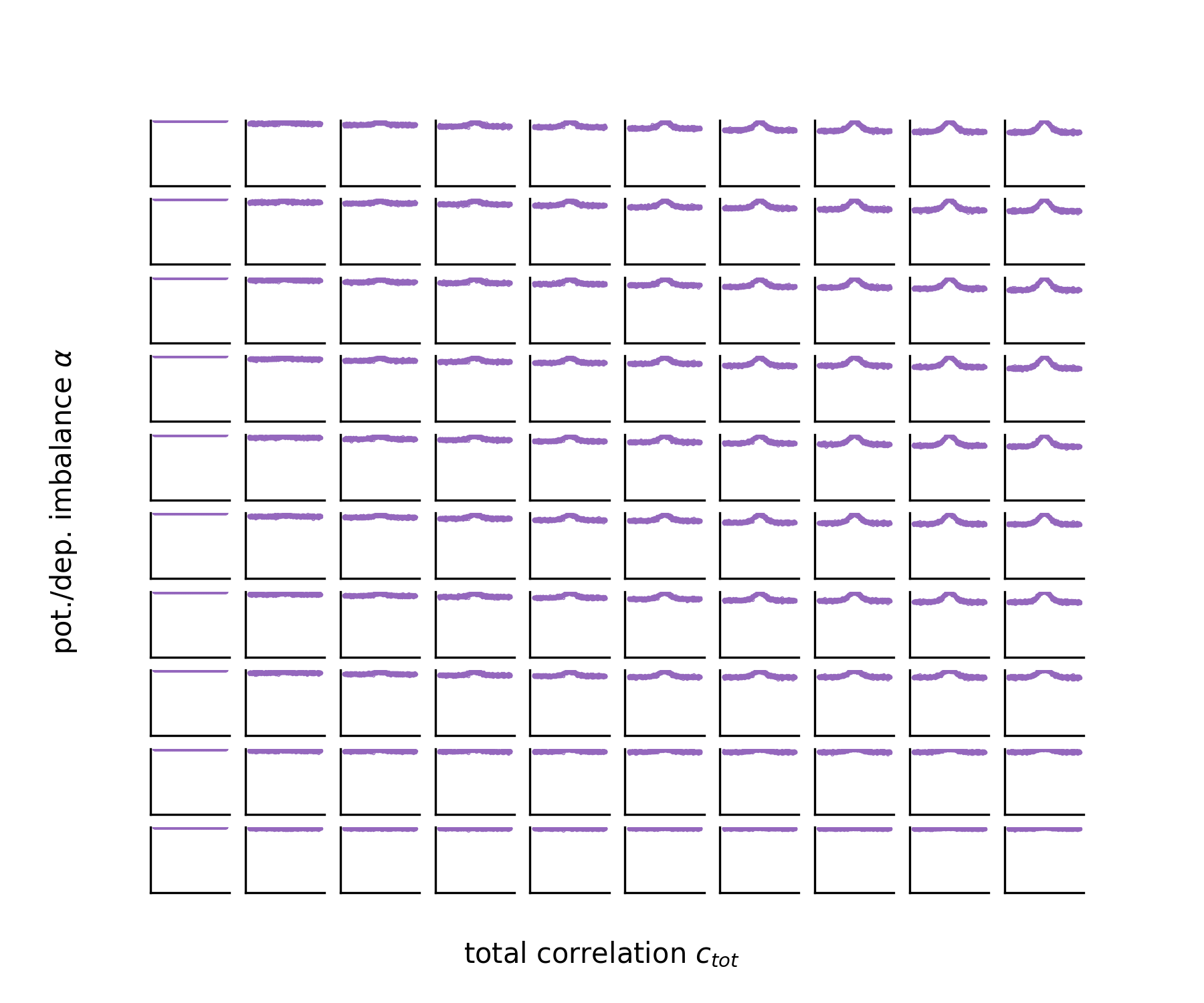

Supplement: S9 Fig — Each subpanel corresponds to each of the 10x10 pixels in the heatmaps shown in Fig 3A3 and 3B3. (TIF) [file pcbi.1012110.s009.tif]

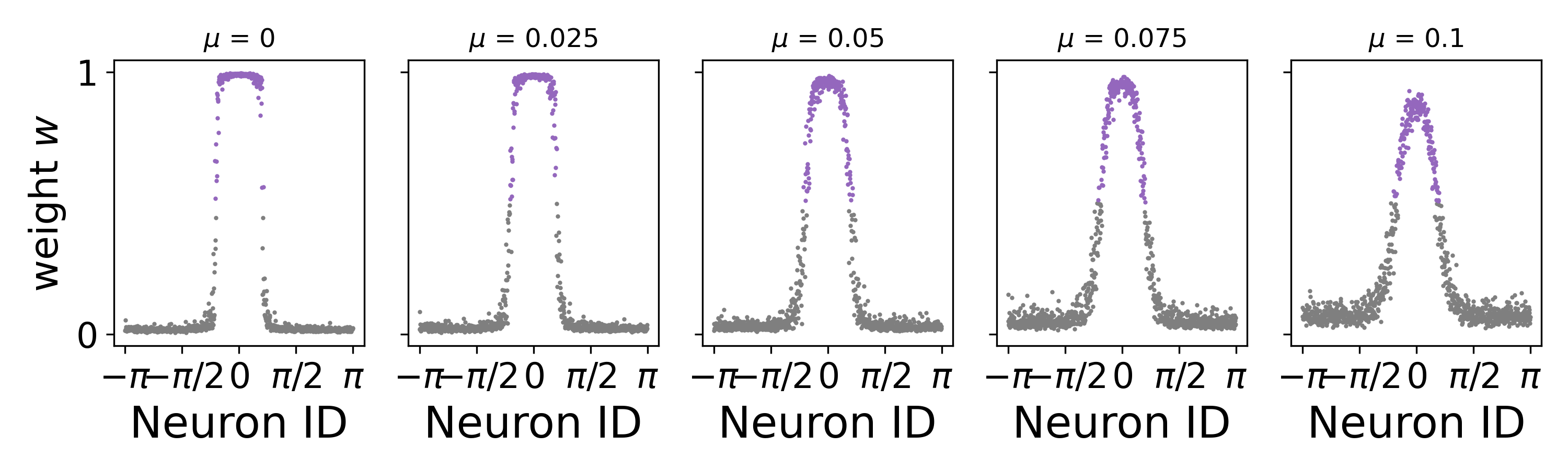

Supplement: S10 Fig — (TIF) [file pcbi.1012110.s010.tif]

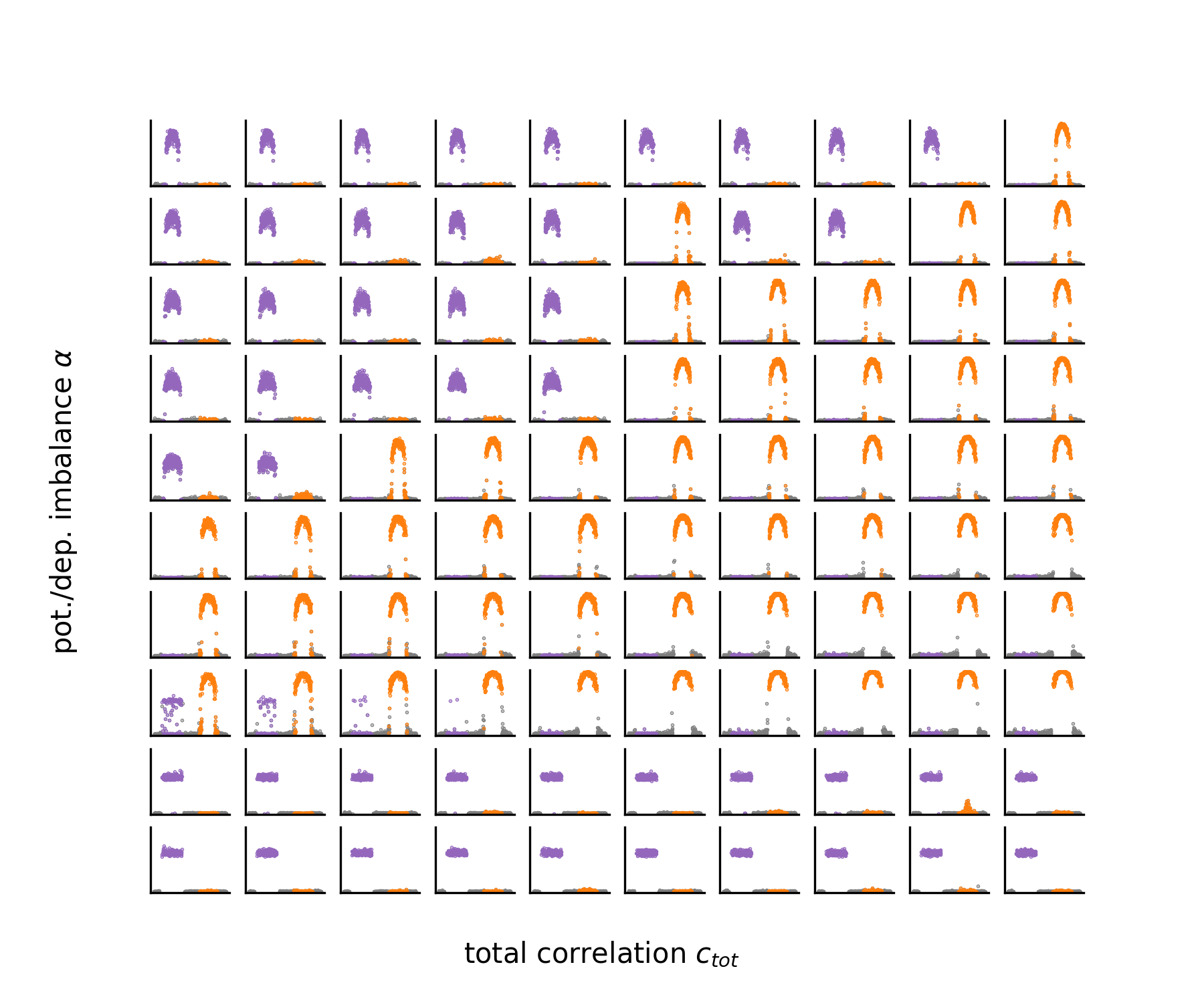

Supplement: S13 Fig — Each subpanel corresponds to each of the 10x10 pixels in the heatmaps shown in Fig 4C1. (TIF) [file pcbi.1012110.s013.tif]

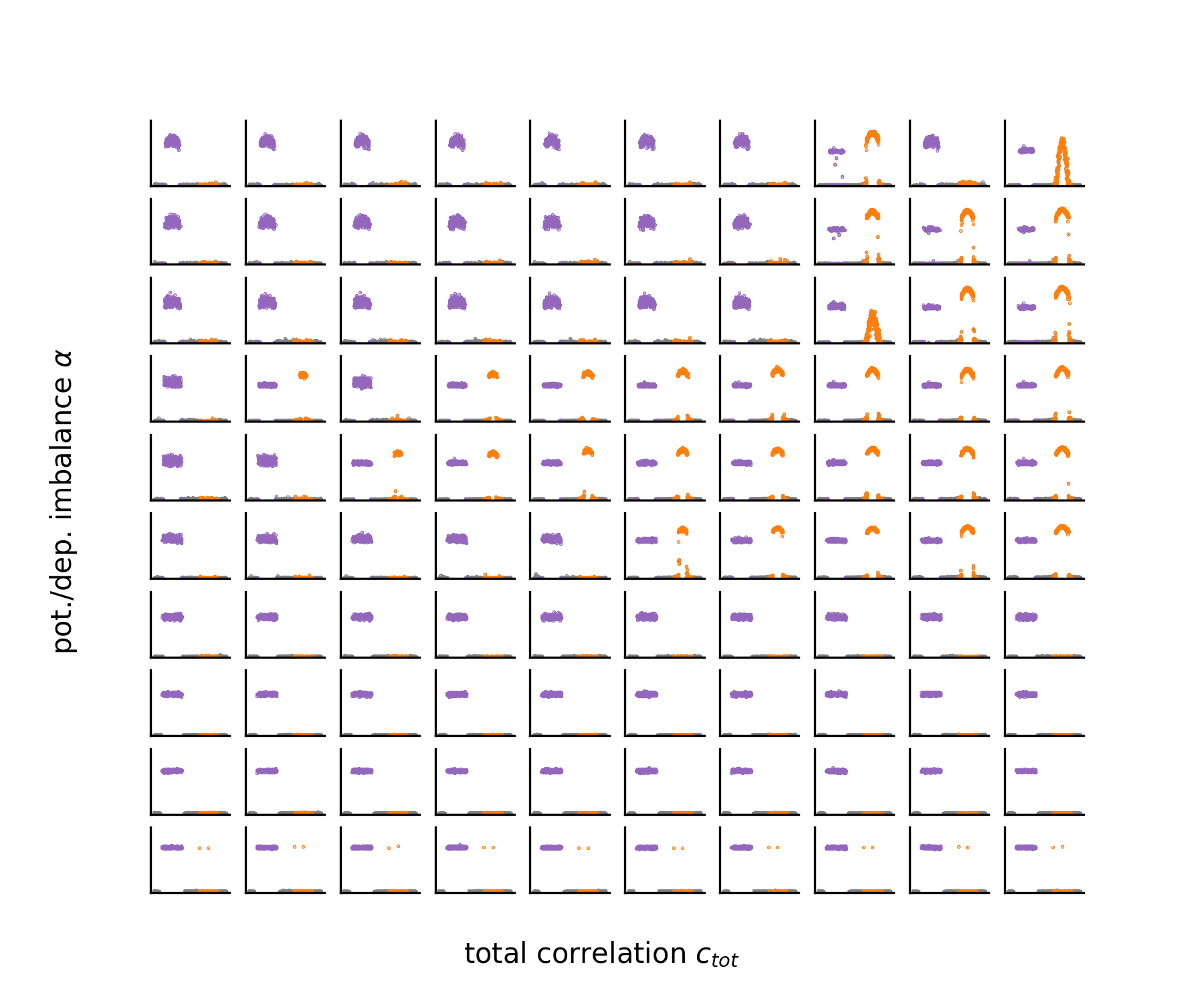

Supplement: S14 Fig — Each subpanel corresponds to each of the 10x10 pixels in the heatmaps shown in Fig 4C2. (TIF) [file pcbi.1012110.s014.tif]

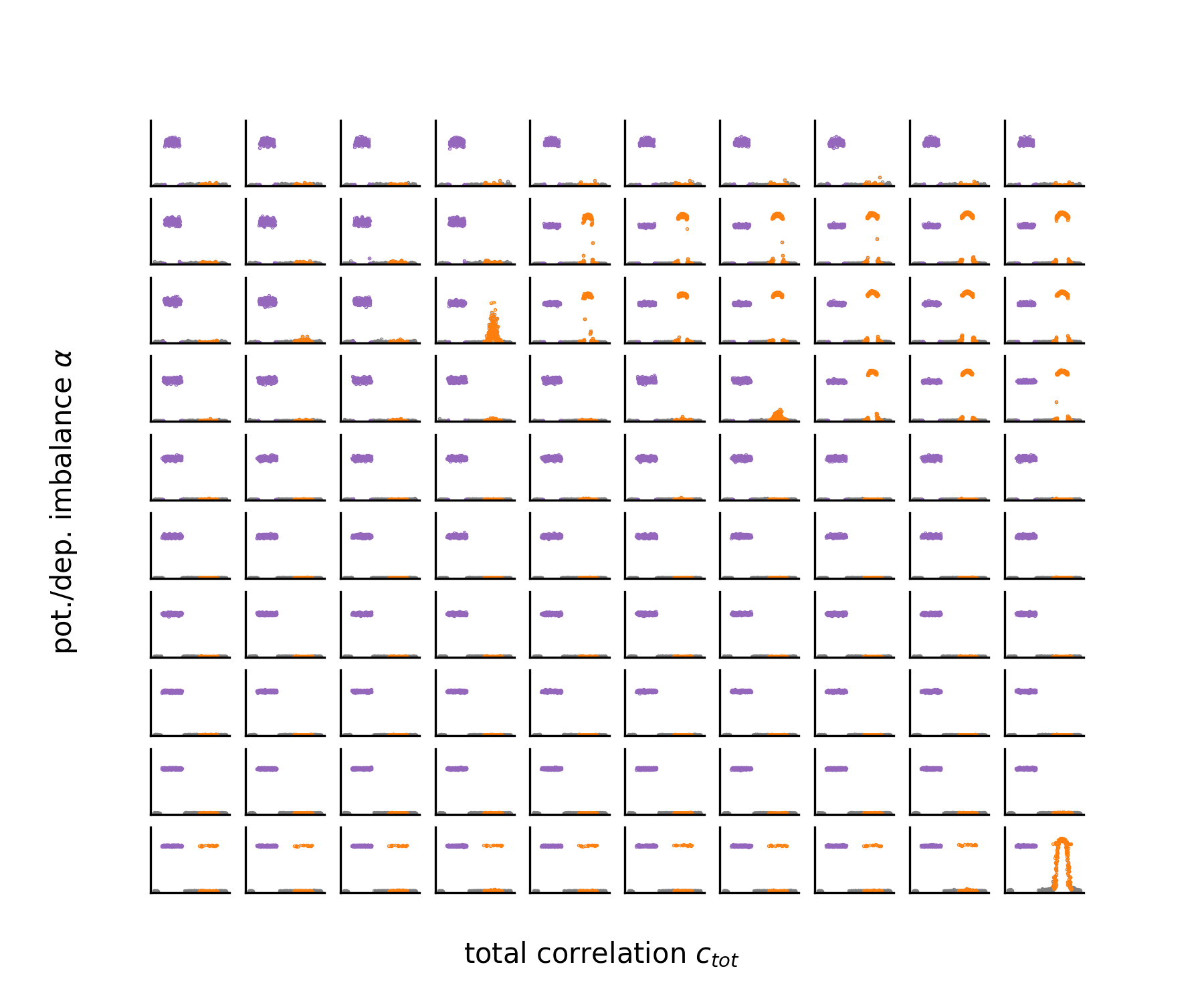

Supplement: S15 Fig — Each subpanel corresponds to each of the 10x10 pixels in the heatmaps shown in Fig 4C3. (TIF) [file pcbi.1012110.s015.tif]

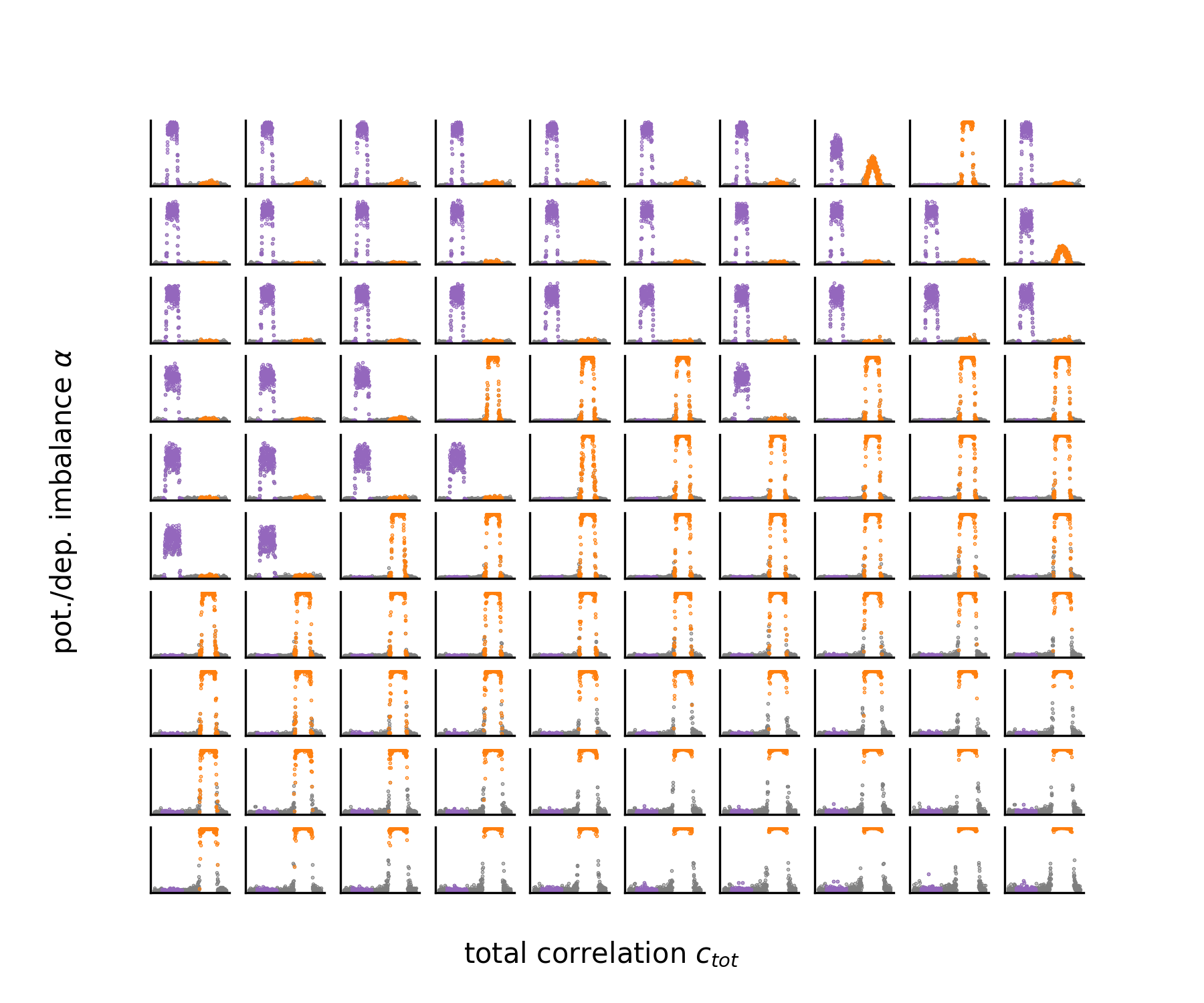

Supplement: S16 Fig — Each subpanel corresponds to each of the 10x10 pixels in the heatmaps shown in Fig 4C4. (TIF) [file pcbi.1012110.s016.tif]
