# Supplementary material for: Learning with filopodia and spines: Complementary strong and weak competition lead to specialized, graded, and protected receptive fields
Source: PLoS Comput Biol. 2024 May 14;20(5):e1012110. doi: 10.1371/journal.pcbi.1012110 (PMC11125506; doi:10.1371/journal.pcbi.1012110)
Supplement: Attachment — Submitted filename: PLOS CB - REB LETTER.pdf [file pcbi.1012110.s017.pdf]

Dear Editor,

We thank the reviewers for their feedback and suggestions. Besides addressing each of their specific points, their overall comments have helped us identify the following in our manuscript:

1. Lack of clarity in the terminology used. We agree that there was an oversimplification of language that blurred the line between each specific learning rule (as defined by the equations), each family of (qualitatively similar) learning rules, and their competition profiles.
2. Unsuccessful conveying of the underlying mechanisms of the model and its connection to other models of STDP via the mean field analysis, for which we also did not present any results beyond Figure 1E.
3. Perspective on the nature of the model, which we agree is better described as a phenomenological proposal than a mechanistic explanation.

For this reason, we have added 9 new supplementary figures and have

1. Added a Table of Terms to help the reader navigate through the terminology used, and changed the latter when appropriate to provide consistent wording. We hope this also helps with the message conveyed in Figure 1. Similarly, we have changed the terminology for filopodia-like and spine-like learning, which we simply call strongly and weakly competitive, instead of additive and multiplicative, as we consider this is less prone to confusions and does not depend on how well FS-STDP approximates these pure forms of learning.
2. Substantially changed the first section. We have changed the text in order to better convey the description of our model, the relation with previous models, and its mean-field analysis. We have also modified Figure 1E, which we consider now provides a more intuitive visualisation of what we meant by filopodia being "effectively additive" and spines "effectively multiplicative".
3. Revised the wording throughout the manuscript to clarify that it is in fact a phenomenological hypothesis of how filopodia and spines could cooperate by incorporating strongly competitive and wealy competitive dynamics (respectively).

We have also made some other modifications that were not explicitly requested by the reviewers, but we considered throughout the process or internal revision:

1. We have standardised parameters across figures in different sections (for example the values c_tot and total time were different in different sections, this did not have any qualitative effect in the results shown).
2. We have changed the parametrization of the mu dependence on the weights, but only implicitly. Instead of fixing a and q directly, we fix new variables mu_filo and mu_spine, and these determine a and q such that the mu fixed points of weights associated with filopodia and spines are similar to the chosen values. This makes model parameters a and q more intuitive to understand (and choose).
3. We have created an online notebook that allows comparing the receptive fields formed with custom correlation structures.

Please also see below our point-by-point response to each of the reviewers. We have adopted the reviewers' suggestions and think we have addressed all of the concerns.

Reviewer #1: *General comment: This paper is very interesting. It extends Gutig et al, 2003 with a time-varying, weight-dependent mu, and it relates the model to recent experimental findings suggesting that weak filopodia synapses are subject to a different learning rule than stronger spine synapses. I really like the concept, and I found the empirical results intuitive given the explanations and visualizations provided. However, the presentation (explanations in text and visualizations in figures) of how the learning rule works and how it produces the empirical results could be improved in order to be digested by a larger audience of theorists and experimentalists.*

We appreciate the feedback from Reviewer 1, which has had a great impact in our revision of the presentation of the model (first section of the results), as well as for the suggestions and typos found. We hope that the mechanisms of the learning rule are clearer and better tailored to the variety of scientific profiles that read the journal.

Suggestions for improvement:
P1L36: *sp - *depolarized*

Changed.

P2L54-59: *What experimental results show unimodal weight distributions? Isn't selectivity typically associated with skewed distributions? Buzsaki, G., Mizuseki, K., 2014. The log-dynamic brain: how skewed distributions affect network operations.*

Nat Rev Neurosci 15, 264–78. doi:10.1038/nrn3687 This study found bimodal distributions:
Dorkenwald, S., Turner, N.L., Macrina, T., Lee, K., Lu, R., Wu, J., Bodor, A.L., Bleckert, A.A., Brittain, D., Kemnitz, N., Silversmith, W.M., Ih, D., Zung, J., Zlateski, A., Tartavull, I., Yu, S.-C., Popovych, S., Wong, W., Castro, M., Jordan, C.S., Wilson, A.M., Froudarakis, E., Buchanan, J., Takeno, M.M., Torres, R., Mahalingam, G., Collman, F., Schneider-Mizell, C.M., Bumbarger, D.J., Li, Y., Becker, L., Suckow, S., Reimer, J., Tolias, A.S., Costa, N.M. da, Reid, R.C., Seung, H.S., 2022. Binary and analog variation of synapses between cortical pyramidal neurons. eLife 11, e76120. doi:10.7554/elife.76120

Thank you very much for referring us to these studies. Here we are finding as a source of selectivity the existence of the spine itself (only correlated enough synapses become spines), so there is no need of having long-tail distributions (as would be the case if all spines are non-zero, and then only the biggest ones dominate, *a single-spike discharge of a presynaptic neuron occasionally evokes a giant depolarization*, as described in *Buzsaki, G., Mizuseki, K.* (2014). This is very related to the following conceptual difference in two different ways of understanding bimodality: (A) bimodality in the distribution of weights, assuming all neurons are connected to all neurons. Here it is clear that neural systems are eminently bimodal in the sense that connectivity is very sparse (even within the local circuit, there is not a high probability of one neuron being connected to another, so most synapses are 0 and then the rest are "non-zero" (they exist)). One could then ignore the zero synapses and study more accurately how spines (structurally unimodal) are distributed. Within those, one could again find bimodality (B), when the distribution of spines is itself bimodal (as they find in Dorkenwald et al., (2022)).
Our study is investigating a strong competition in filopodia as a functional explanation of A, and is less interested in how spines themselves distribute (B). As we mention in the discussion, one could find other ways of modelling how spines learn, we have used nlta-STDP as one classic model but there could be others like log-STDP that would lead to a different type of distribution (in this case log-normal). We have added a clarification on this point in the discussion.

plasticity rule (Knoblauch et al., 2014; Ozcan, 2017). The bimodality observed in filopodia versus spines is

thus, structural, leading to an overall spine sparsity. It should be noted how this is different to a potential bimodality in the distribution of spines itself (as found in Dorkenwald et al. (2022)).

To sum up, here we are focusing on a *first stage of selectivity*, given by sparsity itself, with a simplified picture of how spines are distributed, from which our

model obviates a second stage of specificity given by its skewness.

P3L98-99: *Might be nice to have a Supplementary Figure where you show some example traces of dynamics for a few synaptic weights and their corresponding mu parameter during learning.*

Included (see Figure G2 of the revised manuscript).

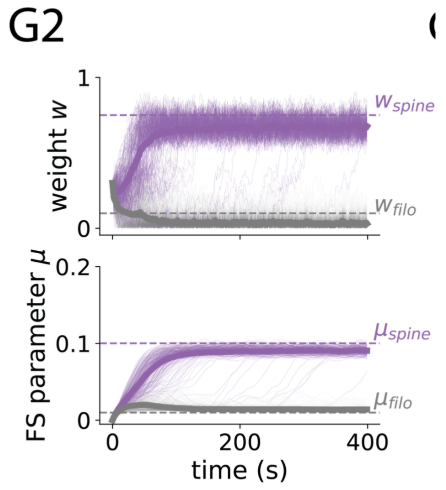

P3L109: *At this point in the text, can you reference where in the equations provided in the methods this competition is implemented? Eq. 3 and 4 describe how one weight evolves independently. Both the potentiation or depression components depend on pre-post correlations, so intuitively, if two presynaptic inputs were correlated with each other, they would share a similar correlation with the output/trace of the postsynaptic neuron, and undergo a similar weight update. However, changing one weight changes the firing rate of the postsynaptic neuron, which then influences other inputs with varying degrees of correlation with each other and with the postsynaptic neuron. This intuition should be provided in the text.*

We have substantially changed the first section with the aim of providing a better intuition of the notions of competition and cooperation between synapses. We address this specific point in the following lines:

terms would be positive, so under equilibrium it is safe to assume that w_i leads to a positive $\Delta f(w_i)$. It should be noted how Equation (6) is a mean-field approximation of STDP, but the notion of competition can also be intuitively extracted from its exact form (see Relevant Equations for STDP in Glossary). If one synapse increases its efficacy, it decreases the average spike-time difference between its pre- and postsynaptic neurons (and increases its own potentiation). However, it also increases the postsynaptic firing rate, which leads to an overall increase of depression for all synapses (assuming depression has a higher amplitude than potentiation). This influence of the total synaptic efficacy on the average depression experienced by all synapses is what the correlation-independent interaction (Equation (6)) represents. On the other hand,

when another presynaptic neuron is temporally correlated with that of a synapse that has been potentiated, the difference in spike times will also (indirectly) decrease. This can compensate the extra depression, and allows the two synapses to cooperate in mutually increasing their weights. This is the effect described by the correlation-dependent interaction (Equation (6)).

Eq. 9 shows that there is a depression component that is independent of cross-correlations. To understand Eq. 9, one needs to find Eq. 12 and 13. The manuscript as is makes it very difficult to find all the information one needs to understand Fig. 1. P5, Fig. 1C-E: I struggled here. To understand how the learning rule in Eq. 3 and 4 relates to the terms "competition," "cooperation," "push," and "pull," I really had to go back and forth between the Eq. 3, 4, 9, 12 and 13, reading the text, and staring at Fig. 1C-E. Something needs to be improved for the naïve reader here.

We hope the vocabulary box, the changes in the text, the additional equations, the changes in Figure 1E overall jointly improve the readability of the text and convey the notions of competition and cooperation in a more understandable manner.

P5, Fig. 1E: What values of the 'a' and 'alpha' parameters do you assume in this panel? Only a large range is given in Table 2.

We apologise for that. The values of "a" were reported in Table 3, but 'alpha' was missing and has now been added to that same table. Note that, there are no multiple 'a' values in Figure 1E anymore, instead "mu_spine" is indicated now.

P5, Fig. 1C-E: There are really two meanings of the word "competition" used in this paper.
The first one is the most important one and relates to the empirical results in Fig. 2E. The end product of an additive STDP rule with weight bound is a binary distribution of weights that do not capture the structure of presynaptic correlations. This is referred to as "strongly competitive." The intuition for this is

that whichever inputs strengthen early, increase the firing rate of the postsynaptic cell, which then increases their correlations with the postsynaptic cell, and they further potentiate. Meanwhile, inputs that are not well correlated with the postsynaptic cell experience a post-synaptic trace-dependent depression (Eq. 3). On the other hand, the end product of a multiplicative STDP rule with weight bound is a unimodal distribution of weights, where the weight co-varies with the degree of correlation with the postsynaptic cell. This is referred to as "weakly competitive" – everyone's allowed a relatively strong weight. However, the baseline weight is elevated, and the signal-to-noise ratio is poor, leading to poor discriminability between stimuli. The hybrid model gets the best of both worlds. It is this meaning of the term "competitive" that is used in Fig. 1C (filopodia are strongly competitive, and spines are weakly competitive). This could be better explained in the text (how to get from Eq. 3 to Eq. 9, 12, 13).

We thank the reviewer for such a great intuitive explanation. We hope this is better explained now in the section "competition profile of FS-STDP", specially in the following paragraph::

> Under this paradigm, the behaviour of a learning rule can be predicted by its *competition profile* (Figure 1E), which is simply the curves of both Δf and f_+ as a function of the synaptic strength. add-STDP is said to induce a *strong competition*, because synapses are split between *winners* and *loosers*, thus following a bimodal distribution. Multiplicative models, like mlt-STDP or mlt/mlt-STDP, induce instead a *weak competition*, where more correlated synapses have bigger synaptic efficacy, but the distribution is rather unimodal. nlta-STDP can have different competition profiles depending on the chosen value of μ, which is why it can lead to both strong and weak competition. In this context, one wonders what is the competition profile of FS-STDP, and how it compares to that of other models of STDP. The answer is that it depends on the synapse you are looking at, and whether it has become a filopodia or a spine. For the μ corresponding to typical synaptic efficacies of filopodia (which usually range from 0 to 0.1), the competition profile observed (grey areas in Figure 1E) are virtually independent of the weight w, being almost exact to add-STDP competition profile (left subpanel). Similarly, we find that for μ corresponding to a synaptic range of w_0 to 1 (range of spines), the competition profile from w_0 to 1 is very similar to nlta-STDP with $\mu = 0.1$ (right subpanel). For this reason, for timescales where μ can be considered constant, changes of filopodia follow strongly competitive dynamics (irrespective of their instantaneous synaptic efficacy), and the same applies for spines and a weak competition.

The second use of the term "competitive" refers to a specific term within Eq. 9 and 12 that falls out of the learning rule (Eq. 3) when firing rate is computed as a weighted sum of inputs. In Figure 1D, this "competitive" force is presented as being "depressive." This caused me as a reader to look at the depression term in Eq. 3, which led to confusion. What's worse is that this "competitive" term in Eq. 9 and 12 can actually switch signs! When it is positive, it is depressing, but when it is negative, it is actually potentiating! I know that this use of the term "competitive" and also the use of the term "cooperative" came directly from Gutig et al., but that doesn't they are best for building intuition about this model! Fig. 1C and 1E present conflicting intuitions due to the dual meaning of the term "competitive". In C, it is

stated that spines (strong weights) "compete" weakly with each other. However, in E, it is shown that the "competitive" term (blue) is stronger for spines! It is only weak for spines close to w0 (in fact it goes negative, turning into "cooperativity"/potentiation). The "cooperative" term (red) is shown as decreasing for weights between w0 to 1. Both of these, at face value, imply that spines are more "competitive"/depressing than filopodia, not less.
The inverse: in C, it is stated that filopodia (weak weights) "compete" strongly with each other. However, in 1E, weights below w0 have strong "cooperation" (red) and low "competition" (blue), so their net effect should be cooperative/potentiating?
I have an even tougher time understanding "push" and "pull," because panels D and E don't show how weak weights influence strong weights or visa versa. Does a weak filopodia "pull" a strong spine towards it, which would be down or depressive? Does a strong spine "push" a weak filopodia away from it, which would also be down/depressive? Please enlighten the reader.
P5, Fig. 1E: Here's a suggestion. What if instead of labeling the two terms in Eq. 9 as "competition" and "cooperation," what if they were instead labeled "correlation-independent interaction" and "correlation-dependent interaction" ? With a sign flip, the correlation-independent interaction could be depressive when that term is negative, and it could be potentiating when that term is positive. Whether or not you adapt this suggestion, I hope you can see that something needs to be made less confusing in Fig. 1 to really grok the key concepts.

We thank the reviewer for this important point. We agree that it was confusing. To address that, regarding the competition and cooperation terms, we have opted for the following: First, given that each of the two terms in the mean-field equation (Eq. 9) have two factors, one that is rule-dependent (and related to the "competition profile" see Table of Terms) and one that specifies the interaction with the rest of synapses (which in one case is correlation-dependent and in the other one is not), we have chosen the following nomenclature, partly inspired by Reviewer 1's proposal:

Correlation-independent interaction → $-\Delta f(w_i)\sum_j w_j$

Competition factor → $\Delta f(w_i)$

Correlation-dependent interaction → $f_+(w_i)\sum_j w_j C^+_{ij}$

Cooperation factor → $f_+(w_i)$

We have considered the following rationale:

- As suggested by Reviewer 1, this nomenclature emphasises that the dynamics are governed by two terms: one that is correlation-independent and one that is correlation-dependent.

- Still, it also highlights the existence of one factor (in each term) that depends on the learning rule choice, and that induces a specific competition-cooperation profile.

- Certainly, it is true that the competition factor does not "always" lead to depression, as can sometimes be negative, overall inducing mean potentiation. This region, however, is intrinsically unstable, as a synapse in that region has necessarily a net positive drive. Thus, it either (1) has enough momentum and/or stochastically accumulates lots of depression in a short period of time to go through it from above, or (2) it will see its efficacy increased until it is allocated in a position where competition cancels cooperation again. This phenomenon is of notable importance to understand the "consolidation" part of the model, the strength of which is governed by parameter "mu_spine" (before by "a").

- We have solved the "exactitude" / "intuitive competition-cooperation based explanation" dilemma by keeping the competition and cooperation factors, as in Gütig (2003), but we have also included in the manuscript a clarification that the correlation-independent term can sometimes actually lead to potentiation.

- Similarly, the word "factor" after competition or cooperation makes the distinction between the high-level phenomena and the mathematical expressions related to each.

P3L125: *Reference Eq. 9.*

Done

P4L160: *I think what you've done here is created presynaptic inputs that have visual receptive fields in orientation space. There is a "reference" input that has a*

von Mises/Gaussian shaped receptive field over orientations. Each input has identical constant firing rates. If each presynaptic neuron is assigned a different preferred orientation, then it will also have a different cross-correlation with the reference neuron. Then in Fig. 2C, I think what you are doing is taking one stimulus orientation, and "showing" it to the network, so that you get a distribution of firing rates over the inputs, and this gets filtered through the learned weights, producing a post-synaptic firing rate for each orientation. It's confusing that the x-axis is labeled "Neuron ID" rather than "Stimulus orientation."

We agree the previous label was confusing, and we incorporate "Stimulus orientation" as suggested.

If this is the case, can you please describe it that way? If not, can you clarify? There is no supplementary section explaining this further. Can you show population heat maps of the presynaptic firing rates versus orientation for two different values of c_total?

The stimulus orientation affects the presynaptic correlation (it corresponds to a shift in that of Figure 2A), but the presynaptic rate is the same for all neurons. We have added a clarification on this in the caption of Figure 2C. Here is the heatmap corresponding to the resulting correlation structure, which now is Supplementary Figure 6.

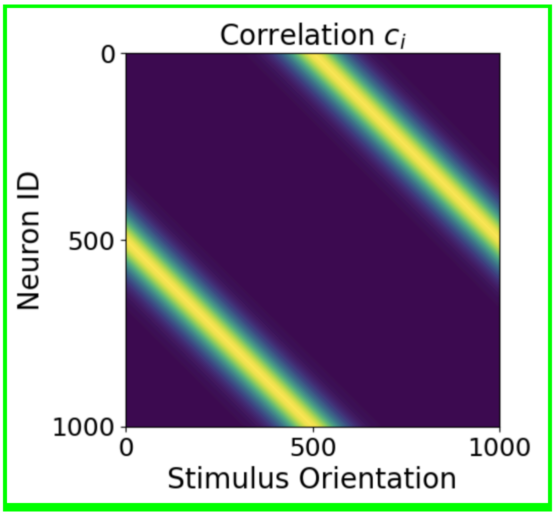

P4L183: *"formation *of the RFs"*

Done

P7, Fig. 2B: “Figure F,” there is no panel F.

Corrected, was Figure 3A (2F in an older version)

P4L165 and P7, Fig. 2B: “discrimination index DI” – should this be reported in panel C? Panel C needs clarification in the legend. Y_pref is not described.

We have defined Y_pref in the figure caption now.

P4L203: *sp - *throw*

Done

P6L215: “**at then*”

Done

P6L233: “*consistently *protects*” ?

Done

P6L234: *There are no blue regions in Fig. 4D. Do you mean grey?*
P6L236: *There are no orange regions in Fig. 4D. Do you mean “changes from red to grey”* ?
P6L243: **orange again*

All corrected

Reviewer #2: *Based on recent experimental observations, the authors developed a computational model implementing a novel synaptic plasticity rule based on STDP-like mechanisms for changes in postsynaptic filopodia (as a structural correlate of silent synapses) and spines (as a structural correlate of non-silent synapses). A major component of this plasticity and learning rule is a strong competition of filopodia to be converted into spines (related to additive plasticity), and a weak competition of spines to encode the representation of input correlations (related to multiplicative plasticity). The filopodium-spine learning rule is elegant because it is formalized using nonlinear-temporally-asymetric learning with just one parameter μ affecting its additivity and multiplicativity (with filopodia/spines having small/large μ values, respectively). Interestingly, the learning rule prevents the disruption of previously learned receptive fields after the emergence of new input correlations - thus supporting memory consolidation. Overall, this computational work represents a solid and innovative contribution to the field.*

We would like to thank Reviewer 2 for their feedback, especially their suggestion of investigating how our model could be affected by intrinsic stochastic processes, and for referring us to recent literature regarding the skewness of distributions.

Comments:

Figure 1G2 and Lines 264-266: 'have excluded the possibility...' and 'imposing a parameterized log dependence such that filopodia follow add-STDP and spines log-STDP'
It is not obvious that a skewed distribution is necessary to represent correlations; it tends to be a conserved feature of synaptic weights and spine sizes (cf. Turrigiano et al., Nature 1998; Hazan & Ziv, J Neurosci 2020).
This point – especially regarding the activity-independence of the skewed weight distribution - could be expanded, particularly given that the distribution of spine weights in Figure 1G2 (but not filopodia weights) does not have the experimentally observed skew with long tails.

This is a very good point. We would like to point out that Figure 1 contains a rather unusual correlation structure (gaussian around a high mean), so there are no inherent “winners” or “highly correlated” subgroups, as is usually the case of study in the literature, or indeed in our von Mises shaped correlations (Figure 2A). We wanted to show that the first stage of the competition is so strong that it

can split correlation structures that are distributed around a single mean (can transform a unimodal correlation structure into a bimodal correlation structure). As for the distribution of spines, FS-STDP induces a (rectified) linear relationship between the correlation value and the final synaptic weight, so in this case one observes a Gaussian reproduction of the original Gaussian (compare spines in Figures 1-G3 and spines in 1-G1). If the correlation itself was long-tailed, as is the case of von-Mises correlations, then one observes this long-tailed distribution of spines (panels G1 (vM) and G3 (vM) included below for completeness, not shown in the paper). As FS-STDP induces a rather linear transformation of correlation into synaptic efficacy, the shape of the distribution of spines is not dependent on the learning rule, but the original shape of the correlation structure.

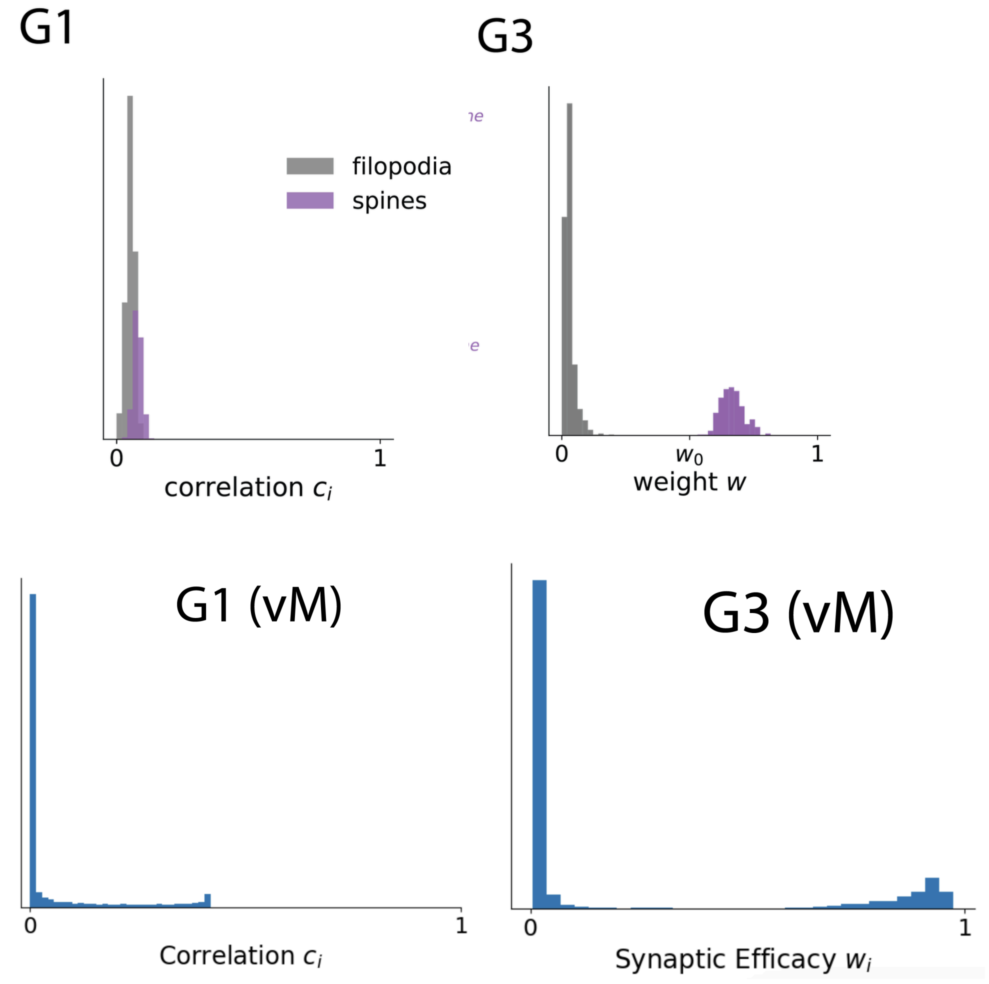

Although the authors mention activity-dependent plasticity rules (e.g. log-STDP) as a potentially relevant mechanism, the lognormal-like distribution of spine sizes (and perhaps also filopodia sizes) emerges even in the complete absence of synaptic transmission– as a result of so-called "intrinsic" (i.e. activity-independent) synaptic dynamics (Hazan & Ziv 2020, Rößler et al. Open Biol 2023). Therefore, recent computational models of lognormal-like (skewed) synaptic weight changes

have been based either on purely intrinsic fluctuations of synapse/spine sizes (Hazan & Ziv 2020, Eggl et al. Comm Biol 2023, Rößler et al. 2023) or on a combination of extrinsic (activity-dependent) and intrinsic dynamics (Rößler et al. 2023) represented as STDP combined with (multiplicative) noise, respectively. How would the inclusion of such "intrinsic" fluctuations in spine (and perhaps filopodia) size affect the main results of the manuscript? Could the authors explore this by including, for example, multiplicative noise as a mechanism for intrinsic synaptic dynamics? If this requires an extensive re-tuning and re-simulation of the model then the authors could at least discuss this in the text (perhaps as an outlook).

That's a great suggestion. We performed extra simulations with multiplicative noise. We have found the effect of multiplicative noise to be mildly affecting the obtained distributions, specially in terms of skewness (computed using scipy.stats.skew), although we find it shifts from a negative to a positive skew as noise increases. In our simulations (see below, not included in the manuscript so far), we added noise at presynaptic term given by: simga_noise*w_i *white_noise, in the presence of a small group of correlated synapses (squared pulse, c_tot = 60)

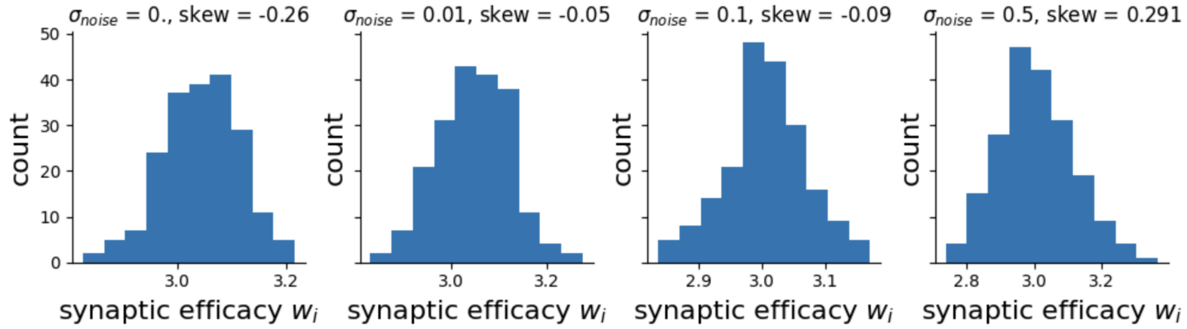

While for spines there are soft, rather than hard-bounds, the bigger the weight is the less potentiation it receives (due to the term (w0^plus - w)). For this reason, while bigger weights experience higher variance due to multiplicative noise, they are also pushed down by the decrease in potentiation.

As mentioned, the main focus of the study was how to allow complementary strong and weak competition in terms of a two stage competition, but the dynamics we have chosen for spines themselves are rather simple and could have many other implementations. If the reviewer or editorial board consider

this is an interesting research line to add to the paper we would happily do that and make a more in-depth study of the effect of intrinsic noise.

Minor:
Line 80 'filpodia'

Done

Line 230 'to take over': 'from taking over'

Done

Line 232 'memory overtake'. 'overwriting' might be clearer.

Done

Line 399 'memory': 'memories'.

Done

Reviewer #3:

Dear Editors,

This manuscript addresses an interesting question: What happens to the learning dynamics within a population of synapses that are undergoing spike-timing dependent plasticity if the weight dependence of synaptic changes, i.e. the update rule (Morrison, 2008) differs between different sub-populations of synapses. Specifically, the manuscript is framed around the question, if state dependent update rules can offer a novel perspective on the long standing trade off between stability and sensitivity in learning. Unfortunately, the present manuscript falls short of providing an answer, that would merit publication in a scientific journal.

We thank Reviewer 3 for such a thorough response that tailors both the details and high-level aspects of our proposed model. Their comments have helped us adopt a more accurate and consistent terminology. In the revision, we don't present our model as one of complementary additive and multiplicative learning, but of strong and weak competition. Then we show that our particular implementation makes filopodia approximate additive STDP and spines approximate nlta with intermediate values of "mu" (which are known to be strongly and weakly competitive, respectively).

Comments of Reviewer 3, although it was not an explicit suggestion, have also inspired us to change the parametrization of the model. Originally, we changed parameter "a" (which shifts the fixed point of "mu" for a given "w") to investigate different degrees of softness in the bounds of filopodia. However, this was inconsistent with our original story, as for "a = 1" one would get that filopodia actually had a similar "mu" to that of spines with "a = 0". For this reason, we now define the parameters "mu_filo" and "mu_spine" and then solve "a" and "q" such that the average "mu" of filopodia and spines is close to that one specified by "mu_filo" and "mu_spine". Now instead of changing a, we change mu_spine, which makes filopodia be consistently following the same type of learning, and only spines have a varying degree of softness in their bounds.

We have also further researched how our model and receptive fields formed compare to "nlta" with intermediate values of "mu", as well as how the mean-field terms of our model compare to that of classic models of STDP, as well as FS-STDP with a constant value of "mu" (which we have called nlta*).

We also agree that “phenomenological proposal” is a better description of our work than “mechanistic explanation”.

Overall, we have specially addressed the following concerns:

- Terminology used and interpretation of previous work
- Validity of the mean-field description used in Gütig (2003) in our model
- Mechanistic vs phenomenological interpretation of this work

We also apologise for the confusions and imprecisions of our original manuscript, which are likely related to many of the concerns raised by Reviewer 3.

The text appears deeply confused about the meaning of additive and multiplicative forms of spike-timing dependent plasticity. In the literature, e.g. Rubin et al. (2001), Guetig et al. (2003), or Morrison et al. (2008) these terms refer to the type of weight dependence realized by the updating functions f_plus(w) and f_minus(w). The choice of no weight dependence, i.e. f_plus(w) = 1 and f_minus(w) = alpha has been termed additive and a linear weight dependence, i.e. f_plus(w) = (1 - w) and f_minus(w) = alpha w (for a lower bound of 0 and an upper bound of 1) has been termed multiplicative. The van Rossum et al. 2001 model implements a mixed form in which f_plus realizes additive and f_minus multiplicative updating. This mixed form is referred to as "additive/multiplicative update rule" in Morrison et al. (2008), a paper which at least according to their reference list, the authors of the present manuscript are familiar with. Nevertheless, in Eq. 7 of the present manuscript, this mixed rule is defined as multiplicative (cf. also the statement: "Two prominent computational models of STDP are additive (add-STDP) (Gerstner et al., 1996; Song et al., 2000) and multiplicative (mult-STDP) (Van Rossum et al., 2000)"). This is also in stark contrast to the terminology used in Guetig et al. (2003) who follow the terminology put forward in Rubin et al. (2001), but whose analyses the present paper heavily relies on.

We are very sorry about the confusion. In order to make the text clearer, we have added a vocabulary box, updated the terminology, and made relevant changes to the introduction and first section of the results (the one that refers to Figure 1).

We would still like to clarify our original wording choice and the motivation behind it.

“mlt-STDP” is defined in the manuscript and it refers to the van Rossum (2001) model. It is not uncommon to use “mlt-STDP” to refer to the van Rossum model (see for example Gilson & Fukai (2011) or Gilson et al (2012)), and we decided to go with this model as a standard choice of multiplicative STDP. We realised our label was not exactly the same as in those studies (“mult” instead of “mlt”) and we have therefore changed it.

Regarding the word “multiplicative” to refer to this last model, we consider that is consistent with that used in Gütig et al (2003):

> *“On the other hand, the multiplicative model* (Kistler and van Hemmen, 2000; *van Rossum et al.,*2000; *Rubin et al.,* 2001) *assumes linear attenuation of potentiating and depressing synaptic changes as the corresponding upper or lower boundary is approached.*”

where the authors themselves are calling multiplicative both “linear/multiplicative” and “purely multiplicative”. The reason for this is, under judgement, that both types of learning are qualitatively equivalent in unimodality, stability, correlation representation, etc It is therefore common to refer to them collectively as “multiplicative”, although we understand that other authors like Morrison et al. (2008) prefer making the distinction explicit. As we have opted to keep “mlt-STDP” for the van Rossum model, we have given the label “mlt/mlt-STDP” to the double weight dependence of Rubin et al. (2001) to avoid any confusion and recognize the existence of these two multiplicative models.

We acknowledge that we originally had taken this abuse of language even one step further, by using terms as “multiplicative-like” or directly “multiplicative learning” to refer to not only “strict multiplicative” and “linear/multiplicative”, but also generalise it to learning regimes (in our learning rule or in nlta) where the competition is weak enough to produce unimodal distributions that continuously match input correlations. While the original intention of this was conveying a simplified picture that aimed at explaining why synapses in our learning rule can find themselves in two regimes that have similarities with either additive or multiplicative STDP, this can also lead to confusion - as the reviewer rightly pointed out- or even inaccuracy, given that the type of receptive

field developed (unimodal / bimodal) can actually even depend on the input statistic, making the same rule with the same parameters potentially be called either “additive” or “multiplicative”. For this reason, and to address the reviewer's concern, we have revised the terminology used and included a vocabulary box that aims to address these grey areas in order to be as exact and consistent as possible. For example, we try to use “weakly competitive” or “soft-bounded” instead of “multiplicative”, as it is less ambiguous.

To make things entirely obscure, the authors state that: "To describe our model mathematically, we make use of nonlinear temporally asymmetric (nlta) STDP (Equation (3) in Methods, (Guetig et al., 2003)). This rule generalizes both add- and mult-STDP via a single parameter mu." It is incomprehensible to me, why the authors would use the same term, "multiplicative" or "mult-STDP" as they call it, to refer to both, the van Rossum et al. (2000) rule and the Guetig et al. (2003) rule (I guess for mu=1). Since does not make any sense (for any value of mu) one worries that the authors have missed one of the key aspects of this family of models.

We hope this is clear now with the changes mentioned above. We also made it clearer that nlta can generalise classic models of STDP (one can recover them with specific mu values), but also adds a new full spectrum associated with mu.

To describe our model mathematically, we make use of a modified version of *nonlinear temporally asymmetric* (nlta)-STDP ((Gütig et al., 2003)):

$$\frac{dw_i(t)}{dt} = \lambda(1 - w_i(t))^{\mu} z_i(t) S_{\text{post}}(t) \quad - \quad \lambda\alpha w_i(t)^{\mu} z_{\text{post}}(t) S_i(t) \tag{1}$$

where w_i is the synaptic efficacy of synapse i, λ the learning rate, and z_i, and z_{post} the pre- and postsynaptic traces, and S_i and S_{post} the pre- and postsynaptic spike trains (see Methods for further definition of these variables). α indicates the imbalance between potentiation and depression for an equivalent spike time difference. In its original form (Equation (1)), nlta-STDP incorporates a parameter μ such that, depending on its value, it induces a stronger or weaker competition between synapses. For example, in the presence of a highly correlated subgroup of synapses, if μ is very small, the steady-state distribution of synaptic weights is bimodal, but as μ increases, the modes of the distribution get closer and closer, resembling what one obtains with mlt-STDP or mlt/mlt-STDP. In particular, for $\mu = 0$ one recovers exactly add-STDP, and for $\mu = 1$, mlt/mlt-STDP. As our model assumes that filopodia follow additive STDP, but spines have soft-bounded weight updates, a simple implementation could be that filopodia follow nlta-STDP with $\mu = 0$, and spines have a μ value that falls within the multiplicative range. However, instead of imposing this in a rule-based manner, we make parameter μ_i (ith synapse) low-pass filter the synaptic efficacy of that synapse w_i

$$\tau_\mu \frac{d\mu_i(t)}{dt} = -\Big(\mu_i(t) - \frac{w_i(t) + a}{q}\Big) \tag{2}$$

Unfortunately, the confusion of the authors does not stop at terminology and model definition but goes to the heart of the learning dynamics, namely the stability of the homogeneous fixed point. The authors claim that the "Add-STDP yields highly selective, bimodal receptive fields, making it a compelling learning rule to uncover

salient statistical patterns in the input structure." I do not understand why the authors say this. In fact, Guetig et al. (2003), i.e. the very reference that this paper's learning rule and analysis seem to be shows based on, precisely that this is not the case (c.f. Guetig et al. 2003, Fig. 7): The additive learning dynamics is so unstable that the emerging bimodal distributions do not capture the correlation structure of the inputs (unless the parameters of the rule, e.g. alpha are fine tuned).

In Guetig et al. 2003, Fig. 7, a comparison between nlta-STDP and add-STDP is made in the context where two correlated subgroups, independent from each other, exist in the input. The authors find that when the strength of the within groups is very small (c = 0.03), in add-STDP the following happens: For one group, all synapses go to the lower bound ("lose" the competition). For the other group, interestingly, a fraction (175/500) reach the upper bound ("win" the competition), while the remaining 325 go to the lower bound, together with the group that has "lost" the competition. In this case, add-STDP is not obtaining a clear winner that unveils all synapses participating in the "salient statistical patterns", but is forced to sample from it. Our intention was to highlight that even though not all correlated synapses win the competition, those winning do reflect (although partially) these salient patterns.

We understand that the original expression could in this sense be considered inaccurate. To address this concern, we have taken our original sentence out and now simply say:

"Add-STDP *yields highly selective, bimodal receptive fields*"

The authors continue by saying: "Mu governs a phase transition such that below a critical value, mu_crit, learning is effectively additive, while for mu > mu_crit learning is qualitatively similar to mult-STDP." Although the authors do not say what they mean by "effectively additive" or "qualitatively similar" this does not seem to make sense. Why should learning become "effectively additive" if the homogeneous fixed point loses stability?

We apologise for the confusion. We have changed it to the following (already shown in a previous paragraph):

> "In its original form (Equation (1)), nlta-STDP incorporates a parameter μ such that, depending on its value, it induces a stronger or weaker competition between synapses. For example, in the presence of a highly

> correlated subgroup of synapses, if μ is very small, the steady-state distribution of synaptic weights is bimodal, but as μ increases, the modes of the distribution get closer and closer, resembling what one obtains with mlt-STDP or mlt/mlt-STDP. In particular, for μ = 0 one recovers exactly add-STDP, and for μ = 1, mlt/mlt-STDP"

Again, the authors seem to miss the core point of having weight dependencies in between additive and multiplicative, i.e. the central contribution of the Guetig et al. (2003) *work. This is most obvious in the presence of correlations, were the value of mu below which the homogeneous fixed point loses (and possibly regains!) stability and the learning converges to bimodal weight distributions, does not have to be small at all and can therefore be far way from an additive update rule (cf. Guetig et al. Fig. 5). In fact, one of the key results of the Guetig et al.* (2003) *work seems to be that intermediate values of mu can compromise between sensitivity and stability, by allowing the synaptic weight distribution to capture the correlation structure of the input while avoiding the instability of the additive model which is more susceptible to the learning rule parameters, such as alpha, than the correlation structure.*

We understand Reviewer 3 is referring to Figure 5B of Gütig et al (2003):

> "*As is evident from Figure 5, for this level of correlation, symmetry breaking occurs below a fairly high value of mu approx* 0.15"

Intermediate values of mu can in fact compromise between sensitivity and stability (see new simulations in Supplementary Figure 9 in the reviewed manuscript):

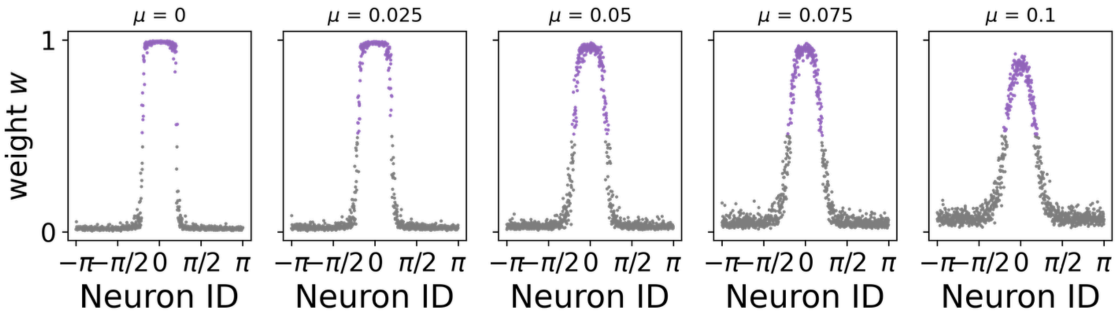

The strength of FS-STDP with respect to intermediate values of mu is that one can have two populations of synapses with very different strengths of

competition. In this sense it is more general as it can have one population approximately follow add-STDP and the other one have an arbitrary value of mu (and hence of competition). This can have an impact when there is a small background correlation instead of having a baseline of 0 correlation. In this scenario FS-STDP behaves differently to an optimised value of “mu” by yielding more selective RFs (see new simulations in Supplementary Figure 12):

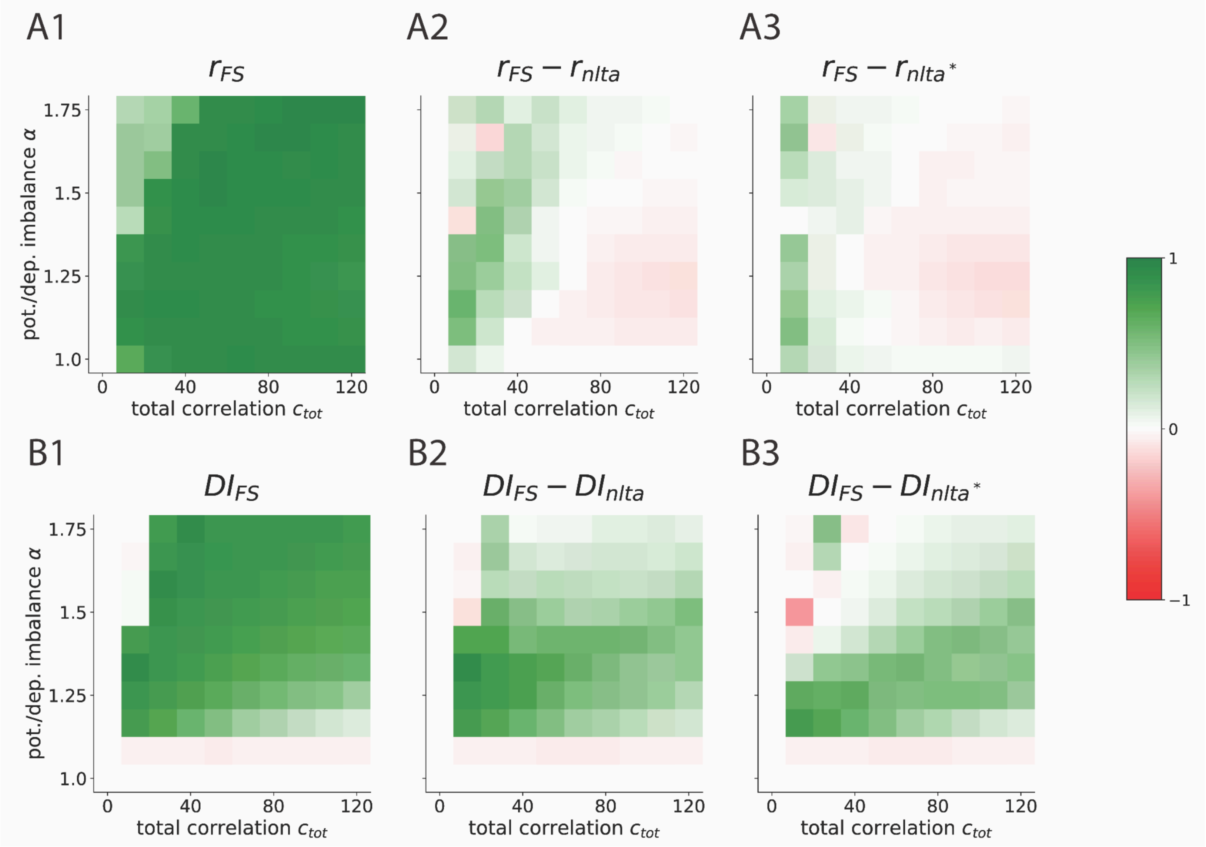

The bizarre confusion between multiplicative, additive and intermediate update rules also extends to the lower right inset of the manuscript's Fig. 1E: According to the text this inset should be showing multiplicative update rules, however it shows neither the Rubin et al. (2001) nor in the van Rossum et al. (2000) models (the two options that the authors have left open to be meant by "mult-STDP").

We apologise again for the abuse of language, which here was actually a mistake. It is indeed not mlt-STDP (where we meant “mlt-like in the sense that it is soft-bounded) but nlta-STDP with mu=0.1. We have corrected this now in Figure 1E.

The main contribution of the present manuscript seem to be two modifications of the Guetig et al. (2003) update rule. Firstly, the parameter mu is made time dependent through a first order differential equation that low pass filters the synaptic weights and whose steady state value (w+a)/q is controlled by two additional parameters. Secondly, the update rule for depression, f_minus is set to the absolute value of the difference between the weight w and a parameter w_0 that defines the boundary between two synaptic states, that the authors associate with filopodia (w < w_0) and spines (w > w_0). Unfortunately, there is very little information on the temporal dynamics of mu and, especially, its interactions with the learning dynamics. The authors neither state the value of the parameter tau_mu nor do they show the time evolution of mu.

We apologise for having forgotten to report the "mu" time constant in Table 2. The value we used throughout all simulations is 20 seconds, and it is now included in that Table. Regardless of that, we have also realised we do not emphasise in the text the motivation or implications of this particular choice, which we now do in the Results when introducing the model.

First of all, we consider "mu" to be an indicator of the "spineness" of a synapse, and it thus involves a biological transformation that cannot be instantaneous. From the experiments of Vardalaki (2022), one can infer this transformation can happen within the order of minutes, but we have decided to make it shorter to speed up simulations. The idea is that transformations of filopodia follow filopodia rules, and transformations of spines, spine rules. Only if the fixed points of those rules would lead to a synaptic value that corresponds to a change in "mu", and therefore a transformation of filopodia to spine (or vice versa), the low-pass filtered value of "mu" would lead to this change.

It is true that this can a priori make the synaptic dynamics quite complex, or even completely different to any of the previous learning rules. However, the filopodia/spine distinction is self-consistent in the sense that the "mu" values of spines lead to synaptic fixed points that correspond to "mu" values of spines, and "mu" values of filopodia lead to synaptic fixed points that correspond to "mu" values of filopodia (if the synaptic distribution matches input correlations, which happens with this learning rule). In particular, we have seen how these changes can be more or less flexibly absorbed depending on the FS parameter "a" (Synaptic Consolidation Section). We consider one of the strengths of our learning rule is precisely that if one assumes filopodia (silent synapses) follow a strong competition, spines (non-silent) follow a weak competition and that the

transition from one to the other is weight-dependent, leading to a stable system in the absence of input perturbations.

Instead, they seem to replace mu(t) by its steady state value (w+a)/q throughout their treatment.

We would like to clarify we only did this to obtain equations (12) and (13) (in the original manuscript), which were then used to produce Figure 1E.

However, we have changed Figure 1E and, instead, we fix “mu” between typical values of filopodia and spines (for each colour). In this sense, what one sees is, assuming decoupled dynamics and fixed mu, what would be the mean-field terms across “w” for typical filopodia and typical spines. We would like to point out that this is relevant because the dynamics undergo a separation of timescales in our model, and probably even more so in biology where the filopodia to spine transition is reported to happen in the order of minutes.

I do not understand why the authors chose to introduce the weight dependence of mu through a separate dynamical equation but then ignore the time dynamics in the rest of the work. Why is an instantaneous weight dependence within f_plus and f_minus not sufficient? I don't mind if it has to be complicated but I think that the reader deserves to know why this is the case. The consequences of having two coupled dynamics should be fully exposed and analysed.

We hope the rationale of this is clear with previous comments and manuscript modifications. To sum up: the choice of “mu” low-pass filtering the synaptic weights is (1) biological : changes from filopodium to spine were observed within minutes, and (2) functional: one would not like noise fluctuations to change the learning rule a synapse is following as (and we understand the concern of Reviewer 3 given that we had not reported the time constant of mu) that this could lead to instabilities and/or oscillations -instead, we would like the learning rule to be dependent on the long-term input statistics. We added this intuition in the manuscript:

was reported to take place within minutes. We use a slightly quicker ($\tau_\mu = 20$ s) time constant to speed-up simulations, which is still considerably slower than synaptic plasticity. Imposing slow dynamics in μ also has a functional motivation, as it makes learning more stable and less prone to oscillatory behaviours, as well as truly dependent on the long-term input statistics rather than instantaneous synaptic states.

The second modification introduced in the manuscript seems to be a stark departure from the intuition (and logic) behind previous studies, where the straightforward motivation for the weight dependence within the update rules (and the parameter mu) was to control the extend to which the upper and lower bounds of the allowed synaptic efficacy range could be felt by the synaptic dynamics within the interval. In the present model, this intuition is only preserved for the updating function f_plus = (1-w)^mu for potentiation which retains the form introduced in Guetig et al. (2003), *such that mu controls the decrease of potentiation as a synapse approaches the upper bound at 1. However, in the modified updating function f_minus, the parameter w_0 introduces an intermediate bound within the allowed synaptic range, which, judging from the weight distributions shown, is still between 0 and 1. According to this term, synapses cannot fall below w_0 from above because the updating rule for depression vanishes at w_0.*

As shown in simulations, the bound imposed by w_0 is "soft", and can be easily trapsassed by step-size effects, specially when the value of "mu_spine" is not very big, and thus the mu corresponding to synapses allocated at w_0 is small enough (see Figures 3-B2 or 3-B3).

For synapses below w_0, the proposed f_minus leads to a strange (from the perspective of hardness of the bounds) behavior of depression: It decreases for synapses that grow towards w_0 from below but increases for synapses that fall towards the lower bound at 0. It seems to me that such a reversed boundary effect should make the learning dynamics below w_0 even more unstable than in the than additive model.

We would like to refer to figure 1E to understand the rationale behind this. Mathematically, our learning rule does contain a region (w_0 - epsilon, w_0 + epsilon) that is unstable, as the only negative contribution of the mean-field term, the "competition", becomes positive, thus driving the synapse to higher synaptic efficacies. This does not lead to instability to "any value smaller than w_0", especially if one considers that the unstable range is itself dependent on "mu" (smaller mu, smaller the range). In Figure 1E, one can see how a synapse allocated at small "w" values would be very indifferent to that instability, keeping rather constant values of f_plus and f_minus across "w".

Biologically, we would like to also rationalise how this could be interpreted for both (1) a filopodia that enters this region from below and (2) a spine that enters it from above. In the case of (1), first of all, and due to the rapid transition that

filopodia exhibit from low to high synaptic efficacy, chances are that by the time the mu value has become big enough as to make that region unstable, the synapse has already escaped it. However, was that not the case, and the synapse allocated in that region for long enough time for mu to change (and “experience” the instability given by the net positive drive), it could be seen as some sort of non-linearity or threshold-based physiological transformation that would favour an extra synaptic strength increase and a transformation from filopodia to spine once a certain synaptic value has been crossed. As for (2), one would need to have a synapse that both has a high mu value (is in the spine regime) and is being pushed to w_0 from above (has small correlations with the rest of spines). Within our framework, the only reason why that would happen is that there has been a change in input correlations, as we study in the last section of the results (Synaptic Consolidation). In that scenario, the hardness of the bound (previously determined by “protection parameter” a, now by “mu_spine”) will determine how likely it is that the synapse stochastically accumulates enough synaptic depression to escape the barrier. In the last section, we make the claim that different hardness in the bounds are not necessarily better or worse, as it can be that (for example under high uncertainty, or when not enough evidence has been accumulated due to immaturity) it is better to have a relatively flexible rule that can easily forget previous input statistics, but in other cases it can be better to make the existent synaptic structure robust.

It is unclear to me how the authors can make such drastic changes to a model but pretend that they can still use an existing analysis. The changes in f_minus and its derivatives as well as the new boundary in the middle of the synaptic range require a re-analysis of the learning dynamics.

Our re-using of previous analysis is backed up by (1) the self-consistency of the learning rule assumptions, (i.e. the synaptic distributions obtained follow learning rules that lead to the same synaptic distributions), which is key to reach an equilibrium, and (2) that these learning rules locally approximate previous learning rules. This second point specifically addresses the “*new boundary*” concern: form the point of view of a “flipodia”, that new bound is non-existent, and from the point of view of a “spine” it would be equivalent to the lower-bound in weakly competing models (mult, mult/mult or nlta with high enough mu).

Inspired by these concerns, we have added Supplementary Figures 1, 2, and 3 where the mean-field terms are explicitly compared for different types of

correlation structures. (Supplementary 3 -with von Mises correlation structures- included here for completeness).

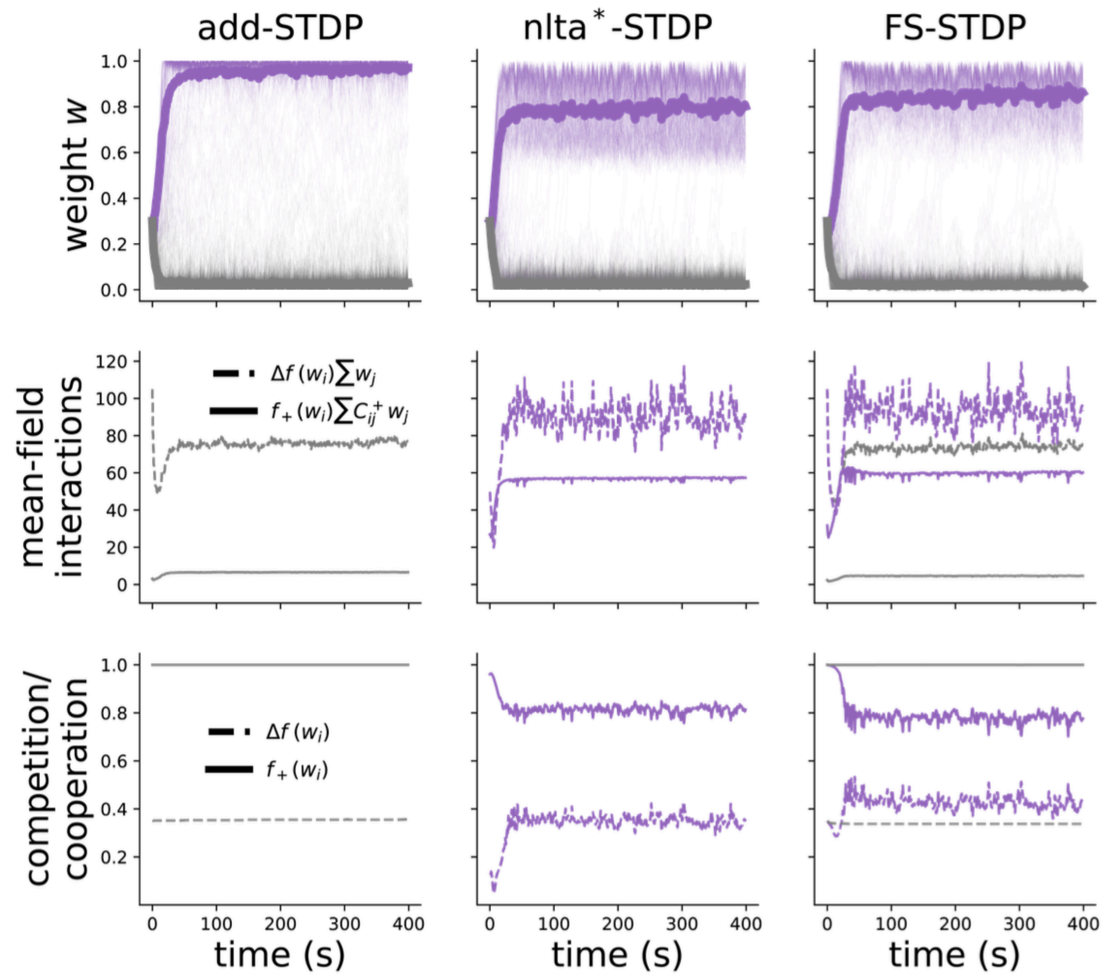

The intention of comparing the competition profiles of our learning rule with that of others was to understand how and why it inherits their properties (which we do find in simulations to happen), so we hope that, while it is only an approximation, simulations can be seen as a support that it is, for what it matters, good enough.

I am not sure what the authors mean by "high-level predictions" when they say: "One of the benefits of using well-established models of learning is that one can make high-level predictions of the learning rule based on previous analyses of these models.", but I am almost certain that this form of "high-level predictions" do not merit publication in an international journal of the format and reach of PLoS Computational Biology.

The idea of this section was to place FS-STDP in context and see how it inherits properties of these well-established models. We have tried to explain its behaviour (which is also observed in simulations in the following sections) in terms of strong and weak competition between synapses, and we have both mathematically and computationally shown when and why our learning rule approximates other existing learning rules (but in a synapse-specific manner).

I am not even speaking of simple concerns such as how synapses can ever cross the w_0 bound from above at all (finite step size?)

Yes, synapses can cross w_0 from above with a finite step size effect, as one would expect from biology, where processes typically have a quantized support.

or how the lower bound is implemented (additive clipping?)

We consider it is a standard practice to implement additive clipping when a synapse is following additive STDP (or approximating it, as we want filopodia to do). We have added details in the Methods section:

> This allows us to explicitly control the strength of the competition of filopodia and spines, by fixing what will be the typical μ values of each type of synapse. Throughout this study, we let $w_0^+ = 1$, and simply write $w_0^- = w_0$. Synaptic efficacies are clipped between $w_{\min} = 0$ and $w_{\max} = 1$. All parameters used in simulations are specified in Table 2.

but about a full analysis of the homogeneous fixed point that depends on the ratio of f_plus and f_minus and whose stability that also depends on the derivative of this ratio.

We hope it is clear again why we have not done a re-analysis, but instead just shown that our synapses can be well approximated by pre-existing learning rules. We acknowledge that the degree of exactitude of the approximation could be debated, but we also consider our work to reflect that it is “at least as good an approximation to reproduce the expected results in simulations”.

In the spirit of comparability to existing work, one also wonders why the present manuscript does not also cover the correlation structures investigated in Guetig et al. (2003) and omits showing the behavior of the modified model in the previously studied scenarios.

We explain this rationale in the text (original manuscript, P4 L160-163). Our first simulations, actually, had a step-shaped correlation structure, where all

correlated synapses had the same value c_i. This was not a good structure to investigate how well our graded synapses can represent those input correlations, and hence we decided to use von Mises shaped correlations. To address the reviewer's point, we have now included two Supplementary Figures (4 and 5) with the receptive fields obtained with such input correlations ("squared pulses"). As expected, the results are very similar to add-STDP, as there is really no difference between spines.

Also, why comparing the novel scenario only to the additive and the van Rossum (?) models and not for instance also the Guetig et al. (2003) model with an optimized fixed value of mu? It seems to me that in most communities this would be standard practice.

This is a great suggestion. Supplementary Figures 9, 10, and 11 address how FS-STDP compares to optimised values of mu in our scenario. Specifically, how when the correlation distribution is less bimodal FS-STDP can help amplify or select a subpopulation of synapses due to its stronger competition at the filopodia level. l

We would also like to point out that the main message of our work is not our specific implementation of the learning rule, but instead studying filopodia and spines as a system with complementary strong and weak competition (we have also changed the title to emphasise this). That is the main reason for the existence of the first section. As we mention in the discussion, there could be other (and possibly better) ways of modelling this, but we consider they will very likely also reproduce the key results of our model as long as they keep some sort of binary distinction between strong and weak competition at each synapse. For example, one could simply incorporate an "if" in the dynamics and make synapses that have a temporally averaged synaptic strength w_0 change from add-STDP to nlta or mlt-STDP (and vice versa). We made the choice of this particular implementation because that "if" is already approximately present in "nlta" via a change in mu, so it seemed as an elegant implementation. We also thought it could be tried to be experimentally connected to some continuous physiological signal that is represented by mu and affects learning dynamics.

At its present level the theoretical contribution of the present manuscript remains very limited and anecdotal.

We hope that the present clarifications, additional simulations, and modifications of the manuscript jointly give now a better support to the main results of our study.

As for biological validity of the presented model, I am astonished by the authors' strong claims. For example, when they state that: "Overall, our results provide a mechanistic explanation of how filopodia and spines could cooperate to overcome the difficulties that these separate forms of learning (additive and multiplicative) each have."

We have discussed this point and agreed that the model is better described as phenomenological than mechanistic, and we have made the corresponding changes in the manuscript.

I don not understand how a phenomenological model like the one presented by the authors can give rise to a mechanistic explanation. Or when they say that: "We have presented a computational model that describes how filopodia and spines are differently affected by plasticity, as well as how they transition from one to the other." Do the authors really mean to imply that referring to synapses below w_0 as filopodia and synapses above w_0 as spines "is a computational model that descripes" how biological filopodia transition to spines and back?

We again agree with Reviewer 3, and have changed the wording of the introduction to make clear that it is a phenomenological proposal and that “how” only refers to within the model, not at the biochemical / molecular level.

In my hands the Vardalaki et al. (2022) work establishes that silent synapses are predominantly located at the tips of filopodia and that these silent synapses can be unsilenced through a spike-timing dependent plasticity protocol. However, since 6 out of 15 of the unsilenced filopodia became longer, in contrast to the 9 that become shorter, it does not seem to be clear cut demonstration for filopodia turning into spines after their efficacies increase, a claim that the authors repeat a total of 5 times throughout their manuscript.

We thank Reviewer 3 for their thorough comments. Again, at the time of writing the manuscript, we made a choice of simplification of the literature to convey a relatively simple story to a broad audience.

As Reviewer 3 points out, only 9 out of the 15 unsilenced filopodia were observed to be shortened (what can be considered a hint of a transformation into a spine). However, then one wonders, if unsilenced filopodia can remain both unsilenced and filopodia, why is that not observed in the distribution of AMPA channels a priori (i.e. why did they not find filopodia with a high synaptic efficacy prior to stimulation?)? We consider that the most plausible explanation is that there is a combination of intrinsic and extrinsic stochastic (but dependent on synaptic efficacy) processes which lead to either an eventual decay back into silent synapses, or a consolidation into a spine. While our model would be rather deterministic in that a synapse that has a consistent synaptic efficacy always is converted into a spine, we don't consider it a stark departure from the experimental results.

Further, the authors imply that a multiplicative model of spine plasticity "is also consistent with Vardalaki et al. (2022), where it was harder to modify the synaptic efficacy of spines compared to filopodia." However, Vardalaki et al. (2022) do not show data on the weight dependence of synaptic updates in spines (nor in filopodia). In fact, in their preparation, spines do not undergo any plasticity under the used spike-timing dependent plasticity protocol, a finding which is inconsistent with the present model, unless one assumes that all spines are already potentiated to their maximally allowed values. Otherwise the failure to induce any plasticity with a perfectly tailored pre- verus post-synaptic activity pattern at an individual synapse is inconsistent with any of the discussed updating rules.

Certainly, one wonders why so well-established experimental results (when it comes to spines) were not reproduced in Varadalaki et al. (2022). We assume there was some experimental variable that was not controlled with respect to classic experimental studies of STDP. For example, visual cortex rarely has changes in input statistics (as opposed to, for instance, hippocampus), and it is likely that those spines had endured further synaptic consolidation processes that prevent them from being easily modified.
However, it seems that within their plasticity.inducing protocol (i) filopodia reacted differently (ii) this difference was related to higher-levels of plasticity, (iii) average synaptic strength of filopodia after potentiation was very similar to that of spines.
We hypothesise this mode of synaptic distributions (shared by spines and potentiated filopodia) could be related to our model's w_0, and that the protocol was strong enough to make filopodia to trespass this wall, but not enough to induce a change in spines. We comment in the discussion that it would be very

beneficial to understand filopodia plasticity to perform experiments in order to exhaustively characterise its dependence on the synaptic state.

I wonder why the authors make no attempt to connect their work to the line of research described in Montgomery et al. (2002), *where a model of different synaptic plasticity states, also involving silent synapses, was proposed already over 20 years ago.*

We thank Reviewer 3 for this suggestion. M*ontgomery et al* (2002) is an experimental paper of hippocampal synapses where one finds state-dependent plasticity too, and is also related to silent synapses. While we cannot extrapolate the results found to our model, which is inspired by experimental results in visual cortex, an area with different plasticity motifs than the hippocampus, the suggested model does indeed have some similarities with ours. We have mentioned it in the discussion.

I also wonder, why the authors remain silent with respect to the biological validity of their model's arguably most unconventional part, namely the form of f_minus. I think that most readers will agree that synaptic efficacies are bounded and that somehow these bounds might be more or less hard. However, a model in which depression accelerates towards the lower bound of 0 but decelerates towards the the intermediate bound of w_0 from above as well as from below will leave many scratching their heads for some sort of guidance on how to think about this postulate.

We would like to recover the argument that this can be better understood from "the perspective" of a filopodia, and then from that of a spine. To start with, a filopodia would transition from 0 to w_0 much more quickly than its mu value to be updated; effectively, that bound does not exist in its dynamics. However, if it did approach this limit from below slow enough as to increase mu and start "feeling that bound from below", we interpreted it as a nonlinear or threshold-crossing effect, in which once it has a specific synaptic efficacy, synaptic efficacy is increased until w_0 is reached. Once it has reached a high synaptic efficacy, and eventually becomes a spine, then the w_0 bound can be simply seen as the classic lower soft-bound of weight-dependent models (perspective of a spine)

I am a big fan of abstract and phenomenological models and I am convinced that their theoretical analyses can have great value in its own right. However, I also

think that over-inflated claims of biological validity, especially in connection with less mature analyses, do more harm than good.

We are very thankful for the constructive criticism received from Reviewer 3. It has helped us identify messages that were ambiguously, mistakenly or non-explicitly conveyed in the original manuscript, and has had a great impact in the way we present both high-level and detailed aspects of the study in the revised manuscript. We are particularly *grateful* for its impact in placing the model in context of past literature of STDP models, in the terminology used, and in its presentation as a phenomenological model, as well as in the presentation of our man-field analysis and the limitations of the model in terms of biological validity.
