# Supplementary material for: Learning with filopodia and spines: Complementary strong and weak competition lead to specialized, graded, and protected receptive fields
Source: PLoS Comput Biol. 2024 May 14;20(5):e1012110. doi: 10.1371/journal.pcbi.1012110 (PMC11125506; doi:10.1371/journal.pcbi.1012110)
Supplement: Attachment — Submitted filename: PLOS CB - REB LETTER 2.pdf [file pcbi.1012110.s018.pdf]

Dear Editor,

We thank the reviewers for taking the time to review the revised version of the manuscript, as well for their new feedback.

Please also see below our point-by-point response to each of the reviewers. We have adopted the reviewers' new suggestions and hope the manuscript is now fit for publication.

Reviewer #1: The authors have addressed my previous concerns. Below are some additional minor issues that should be corrected, but the manuscript is otherwise ready for publication.

P3L131 – typo: "in the of weight dependence"

Corrected.

P5L148 – not sure if Eq. (2) needs to be repeated as Eq. (5)

Done.

P7, Fig. 1E legend – are the grey shaded areas for u in [0, 0.1], and the purple areas for u in [0.5, 1] ? Please indicate this in the legend.

Done.

P8L16 – do you mean mu_spine here?

Yes! Corrected.

P9, Fig. 2 – In the previous review, I asked for clarification about the stimulus. I understand it better now (tell me if I'm wrong). All units are firing at the same time-averaged rate. Some units are highly correlated with a reference spike train (von Mises theta = 0), and others have correlations that drop off with increasing von Mises angle theta. So it is definitely not like an oriented bar visual stimulus being presented, where different neurons would have different degrees of correlation because they have different "orientation preferences". I know I requested the xlabel "Stimulus Orientation" for Fig. 2C, but now that I understand that it is the correlations of the inputs that are being rotated, perhaps a more precise xlabel would be "Rotation of Input Correlations (theta)".

Done.

P11L382 – *typo: "biomdally"*

Corrected.

P12, *Fig. 4D – For the grey regions where memory overlap is allowed, wouldn't the discriminability of A vs. B be poor? I guess the postsynaptic cell would now be an "A or B" detector, and still discriminate vs. other correlation rotations? Ideally this would be discussed in the paper.*

Done:

the correlation structure from pattern A to pattern B for 400 seconds more. Applying this protocol, one can encounter three different scenarios: (i) the previous memory is erased with the new pattern taking over (Figure 4B2, *Total Overwriting*), (ii) the previous memory is maintained, but pattern B is also imprinted (the neuron becomes an *A or B* detector, Figure 4B3, *Partial Overwriting*), and (iii) the previous memory is maintained, and the new pattern is not imprinted (Figure 4B4, *No Overwriting*). We classify the trajectories

P13L401 – *grammar: "high values [of] mu"*

Corrected.

P14L466 – *typo: "small(s)"*

Corrected.

P14L468 – *one of these should be mu_filo*

Corrected.

P15, *Table 2: mu_spine is labeled mu_filo*

Corrected.

P17L521 – *typo: "(or) preferred angle"*

Corrected.

Reviewer #2: I think the authors have addressed the main points of the reviewers.

Authors should make the computational code underlying their results fully available. I did not notice this in the revised manuscript. Please add the link or repository where the code is uploaded.

Sorry if this was not clear, there is a section of Code Availability after Methods. Code for reproduction can be found at
https://github.com/albesagonzalez/filopodium-spine-learning

Minor point: I know the authors present their plasticity rule as a phenomenological model rather than a mechanistic model, but if they have an idea of what "some continuous physiological signal represented by mu" might be, they might want to mention it in a sentence in the Discussion.

We have mentioned a note on this in the discussion:

& Ramaswami, 2012). Finally, we leave open the question of what physiological signal(s) could represent a temporally averaged version of the synaptic strength, which in our model is called μ. As we interpret synaptic strength as the amount of AMPA receptors at the postsynaptic level, variable μ could depend on retrograde signals, which can play an important role in synaptic maturation (Fitzsimonds & Poo, 1998). However, it could also be dependent on other locally available signals such as synaptic volume.
